# Heterogeneous cardiac sympathetic innervation gradients promote arrhythmogenesis in murine dilated cardiomyopathy

**DOI:** 10.1172/jci.insight.157956

**Published:** 2023-11-22

**Authors:** Al-Hassan J. Dajani, Michael B. Liu, Michael A. Olaopa, Lucian Cao, Carla Valenzuela-Ripoll, Timothy J. Davis, Megan D. Poston, Elizabeth H. Smith, Jaime Contreras, Marissa Pennino, Christopher M. Waldmann, Donald B. Hoover, Jason T. Lee, Patrick Y. Jay, Ali Javaheri, Roger Slavik, Zhilin Qu, Olujimi A. Ajijola

**Affiliations:** 1 UCLA Cardiac Arrhythmia Center, UCLA Neurocardiology Research Program of Excellence, and Department of Medicine, UCLA, Los Angeles, California, USA.; 2 Washington University School of Medicine, St. Louis, Missouri, USA.; 3 Department of Biomedical Sciences, Quillen College of Medicine, and; 4 Center of Excellence in Inflammation, Infectious Disease and Immunity, East Tennessee State University, Johnson City, Tennessee, USA.; 5 Ahmanson Translational Theranostics Division, Department of Molecular and Medical Pharmacology, David Geffen School of Medicine at UCLA, Los Angeles, California, USA.; 6 Department of Nuclear Medicine, University Medical Center of the Johannes Gutenberg-University, Mainz, Germany.; 7 Crump Institute for Molecular Imaging, Department of Molecular and Medical Pharmacology, David Geffen School of Medicine at UCLA, Los Angeles, California, USA.; 8 Molecular Imaging Program at Stanford, Department of Radiology, Stanford University School of Medicine, Stanford, California, USA.; 9 Alnylam Pharmaceuticals, Cambridge, Massachusetts, USA.; 10 John J. Cochran Veterans Hospital, St. Louis, Missouri, USA.

**Keywords:** Cardiology, Arrhythmias, Heart failure, Neurodegeneration

## Abstract

Ventricular arrhythmias (VAs) in heart failure are enhanced by sympathoexcitation. However, radiotracer studies of catecholamine uptake in failing human hearts demonstrate a proclivity for VAs in patients with reduced cardiac sympathetic innervation. We hypothesized that this counterintuitive finding is explained by heterogeneous loss of sympathetic nerves in the failing heart. In a murine model of dilated cardiomyopathy (DCM), delayed PET imaging of sympathetic nerve density using the catecholamine analog [^11^C]meta-Hydroxyephedrine demonstrated global hypoinnervation in ventricular myocardium. Although reduced, sympathetic innervation in 2 distinct DCM models invariably exhibited transmural (epicardial to endocardial) gradients, with the endocardium being devoid of sympathetic nerve fibers versus controls. Further, the severity of transmural innervation gradients was correlated with VAs. Transmural innervation gradients were also identified in human left ventricular free wall samples from DCM versus controls. We investigated mechanisms underlying this relationship by in silico studies in 1D, 2D, and 3D models of failing and normal human hearts, finding that arrhythmogenesis increased as heterogeneity in sympathetic innervation worsened. Specifically, both DCM-induced myocyte electrical remodeling and spatially inhomogeneous innervation gradients synergistically worsened arrhythmogenesis. Thus, heterogeneous innervation gradients in DCM promoted arrhythmogenesis. Restoration of homogeneous sympathetic innervation in the failing heart may reduce VAs.

## Introduction

Ventricular arrhythmias (VAs) remain a leading cause of death in nonischemic dilated cardiomyopathy (DCM) (1). The autonomic nervous system (ANS), through its parasympathetic and sympathetic divisions, extensively innervates the heart and is an important modulator of cardiac electrophysiology (2, 3). Dysfunction within the ANS, specifically increased cardiac sympathetic tone and withdrawal of parasympathetic signaling, has been linked to arrhythmogenesis (4). The role of sympathetic signaling in promoting cardiac dysfunction and arrhythmias is underscored by the strong clinical data supporting the use of β-adrenergic receptor blockers (5–7) and other forms of neurohormonal blockade in chronic DCM. Further, nonpharmacologic antiadrenergic therapies such as bilateral cardiac sympathetic denervation, stellate ganglion blockade using anesthetic agents, and other neural interventions in the setting of cardiac dysfunction suppress VAs. These interventions physically or functionally interrupt signaling from sympathetic neurons and the fibers directly innervating the heart, distinct from circulating catecholamines from the adrenal glands.

Paradoxically, while congestive heart failure (CHF), whether ischemic or DCM, is characterized by chronic sympathetic excess, it is associated with reduced cardiac sympathetic innervation in humans (8) and in canine (9) and rodent (10) experimental models of CHF. This finding has been mechanistically related to reduced expression of the neurotrophin nerve growth factor (*Ngf*) and increased production of the neurorepellant semaphorin 3A (*Sema3a*) by cardiomyocytes in the failing heart (11–13). The detrimental consequence of reduced sympathetic innervation was clinically demonstrated in the ADMIRE-HF study (14), using delayed functional imaging of cardiac sympathetic nerves with a radiolabeled tracer, iodine-123 meta-iodobenzylguanidine ([^123^I]-mIBG). In this study, reduced sympathetic innervation, identified by lower heart/mediastinal uptake of [^123^I]-mIBG, was associated with higher risk of severe VAs, heart failure hospitalization, and mortality.

However, the mechanisms by which loss of sympathetic innervation in chronic CHF enhances arrhythmogenesis remain poorly understood. We hypothesized that 1) the loss of sympathetic nerves in the failing heart is heterogeneous and 2) this heterogeneity permits the emergence of dynamic substrates that permit reentry and the triggers that initiate them during sympathetic activation. The goal of the present study was to test this hypothesis in an established mouse model of nonischemic DCM (DCM Tg9) (15–17). This model exhibits similar traits to human heart failure (15) without the potential confounding factors associated with surgical models (e.g., coronary artery ligation) and thus is an ideal model to study innervation in an unperturbed fashion.

We demonstrate that progression of DCM increases susceptibility to ventricular arrhythmogenesis. Importantly, we correlate this susceptibility to an increased transmural gradient of sympathetic innervation. Finally, we show how the heterogeneity in sympathetic innervation gradients promotes arrhythmogenesis using in silico models of normal and failing human myocardium and heart. This study provides what we believe are novel insights into how neural remodeling in nonischemic heart failure promotes ventricular arrhythmogenesis.

## Results

### DCM Tg9 model recapitulates human heart failure and demonstrates decreased functional sympathetic innervation.

**Figure 1:**
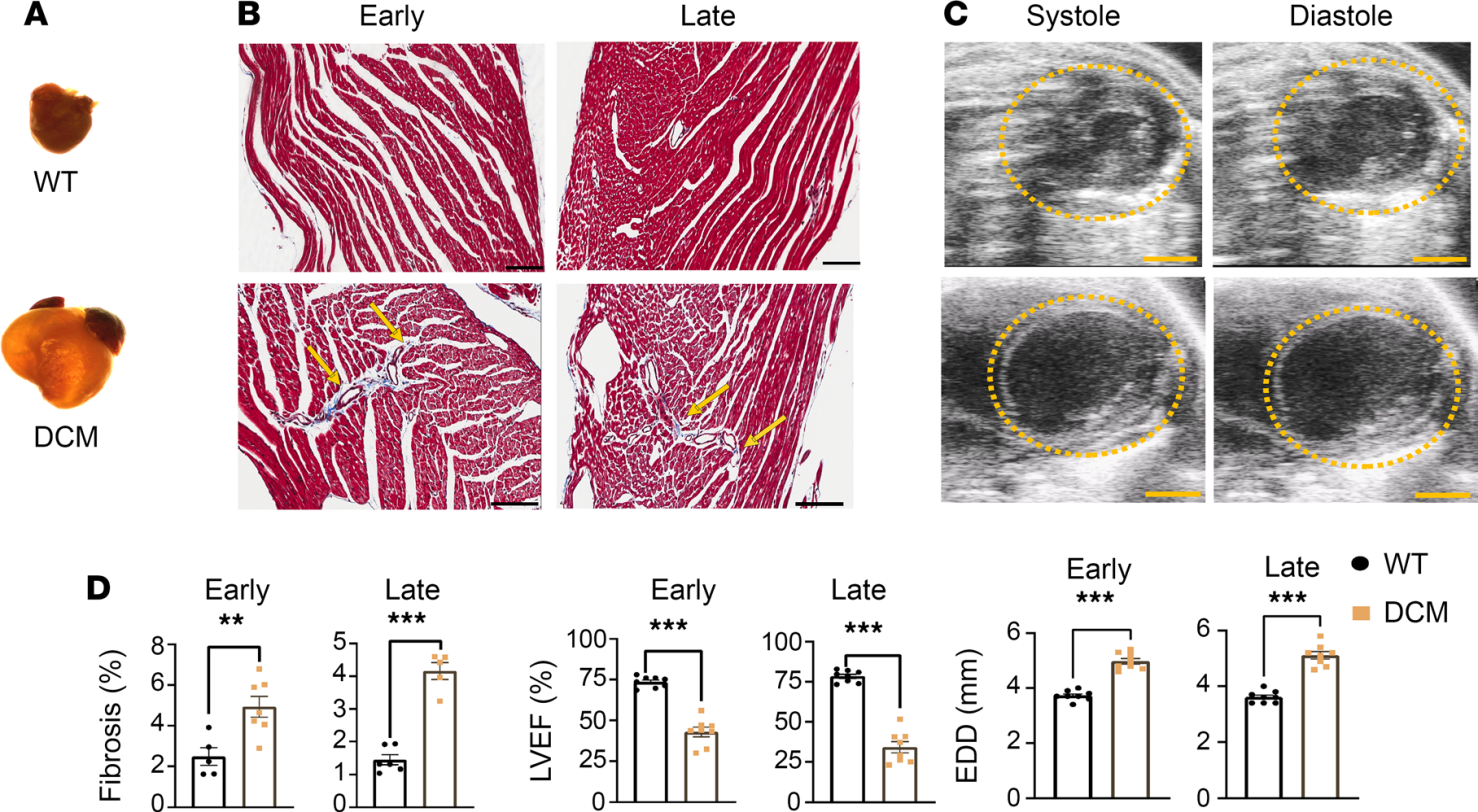
Transgenic DCM mouse model recapitulates human heart failure. (**A**) Control (top row) and DCM (bottom row) mouse hearts imaged using a light microscope. (**B**) Digitally scanned images of transmural myocardial sections from control and DCM mice in early (left) and late (right) stages stained with Masson’s trichrome to indicate fibrosis. Image scale bars are 100 μm. (**C**) Images of left ventricular end systolic diameter (ESD, left) and end diastolic diameter (EDD, right) taken using echocardiography for control and DCM mice. Image scale bars are 2 mm. (**D**) Fibrosis levels in WT vs. DCM in myocardium of left ventricle in early (left, *n* = 5 for control, *n* = 7 for DCM, **P = 0.0045, Shapiro-Wilk test, Welch’s *t* test) and late stages (right, *n* = 6 for control, *n* = 5 DCM, ***P < 0.0001, Shapiro-Wilk test, Welch’s *t* test). LVEF (%) in control and DCM mice in early (left, *n* = 8 for control, *n* = 8 for DCM, ***P < 0.0001, Shapiro-Wilk test, Welch’s *t* test) and late stages (right, *n* = 8 for control, *n* = 8 for DCM, ***P < 0.0001, Shapiro-Wilk test, Welch’s *t* test). EDD in WT vs. DCM mice in early (left, *n* = 8 for control, *n* = 8 for DCM, ***P < 0.0001, Shapiro-Wilk test, Welch’s *t* test) and late stages (right, *n* = 8 for control, *n* = 8 for DCM, ***P < 0.0001, Shapiro-Wilk test, Welch’s *t* test). “Early” refers to mice less than or equal to 8 weeks of age, while “late” refers to mice older than 8 weeks.

First, we confirmed that the DCM mouse model exhibits features of human heart failure, particularly differential changes in functional sympathetic innervation as assessed by delayed PET imaging. DCM mice exhibited cardiomegaly compared with controls (Figure 1A), progressive interstitial fibrosis (Figure 1, B and D), and on echocardiography, progressive decline in left ventricular ejection fraction (LVEF) and increased left ventricular EDD (Figure 1, C and D).

**Figure 2:**
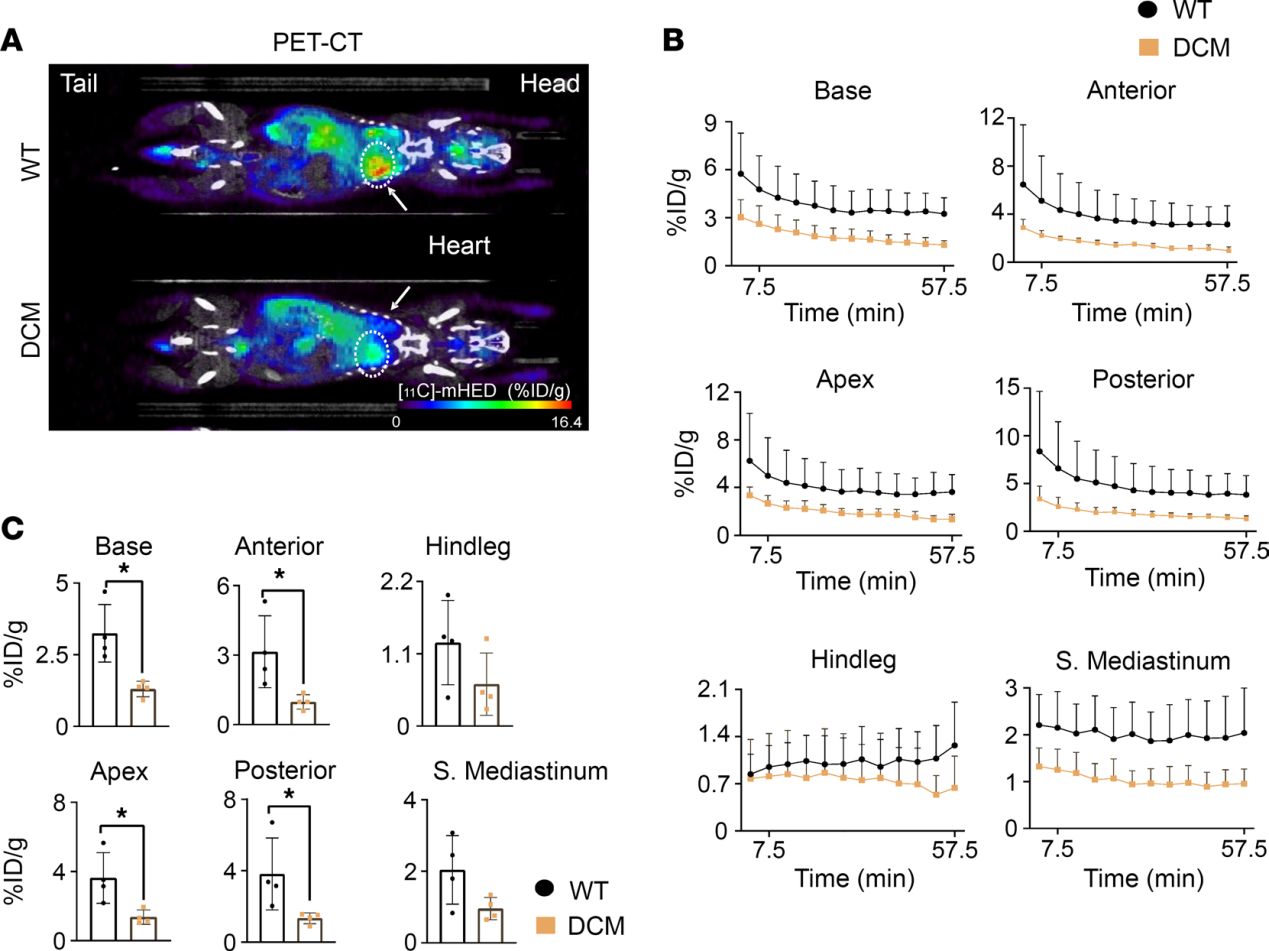
DCM model shows decreased cardiac sympathetic innervation. (**A**) Representative [^11^C]meta-Hydroxyephedrine PET-CT images taken at the 60-minute time point of WT (top) and DCM (bottom) mouse models scaled to units of percentage injected dose per gram of tissue (% ID/g) (head, heart, and tail labeled for orientation). (**B**) [^11^C]meta-Hydroxyephedrine time-activity curves (0–60 minutes) showing uptake in adrenergic nerve terminals of various tissues in control and DCM mice. (**C**) Quantification at 60 minutes to show uptake of [^11^C]meta-Hydroxyephedrine in adrenergic nerve terminals of various tissues in control and DCM mice: cardiac base (*n* = 4 for control, *n* = 4 for DCM, *P = 0.0286, Mann-Whitney test), cardiac apex (*n* = 4 for control, *n* = 4 for DCM, *P = 0.0286, Mann-Whitney test), cardiac ant. wall (*n* = 4 for control, *n* = 4 for DCM, *P = 0.0286, Mann-Whitney test), cardiac post. wall (*n* = 4 for control, *n* = 4 for DCM, *P = 0.0286, Mann-Whitney test), hind leg (*n* = 4 for control, *n* = 4 for DCM, *P* = 0.3429, Mann-Whitney test), and superior mediastinum (*n* = 4 for control, *n* = 4 for DCM, *P* = 0.1143, Mann-Whitney test).

Next, [^11^C]meta-Hydroxyephedrine ([^11^C]-mHED) PET-CT was performed to quantify functional sympathetic cardiac innervation on delayed imaging in DCM and control mice. To validate the synthesis of [^11^C]-mHED and its specificity for sympathetic nerve endings, delayed PET-CT imaging was performed following depletion of cardiac sympathetic nerves using 6-hydroxydopamine (6-OHDA). As shown in Supplemental Figure 1, A–D (supplemental material available online with this article; https://doi.org/10.1172/jci.insight.157956DS1), mice treated with 6-OHDA demonstrated little to no sympathetic innervation on immunohistochemistry. Consistent with this, PET-CT imaging using [^11^C]-mHED demonstrated severely reduced uptake in the heart but not hind leg or mediastinum, where sympathetic innervation is lower. When performed in late-stage DCM versus age-matched control littermates, noncardiac tissues, including liver and hind leg muscle, were not significantly different in radioactivity level. However, the base, apex, anterior wall, and posterior wall of the heart showed significantly less radioactivity in DCM mice compared with control mice (Figure 2), indicating that sympathetic innervation is reduced structurally and functionally in DCM mice.

### DCM is characterized by spatially heterogeneous cardiac sympathetic innervation.

**Figure 3:**
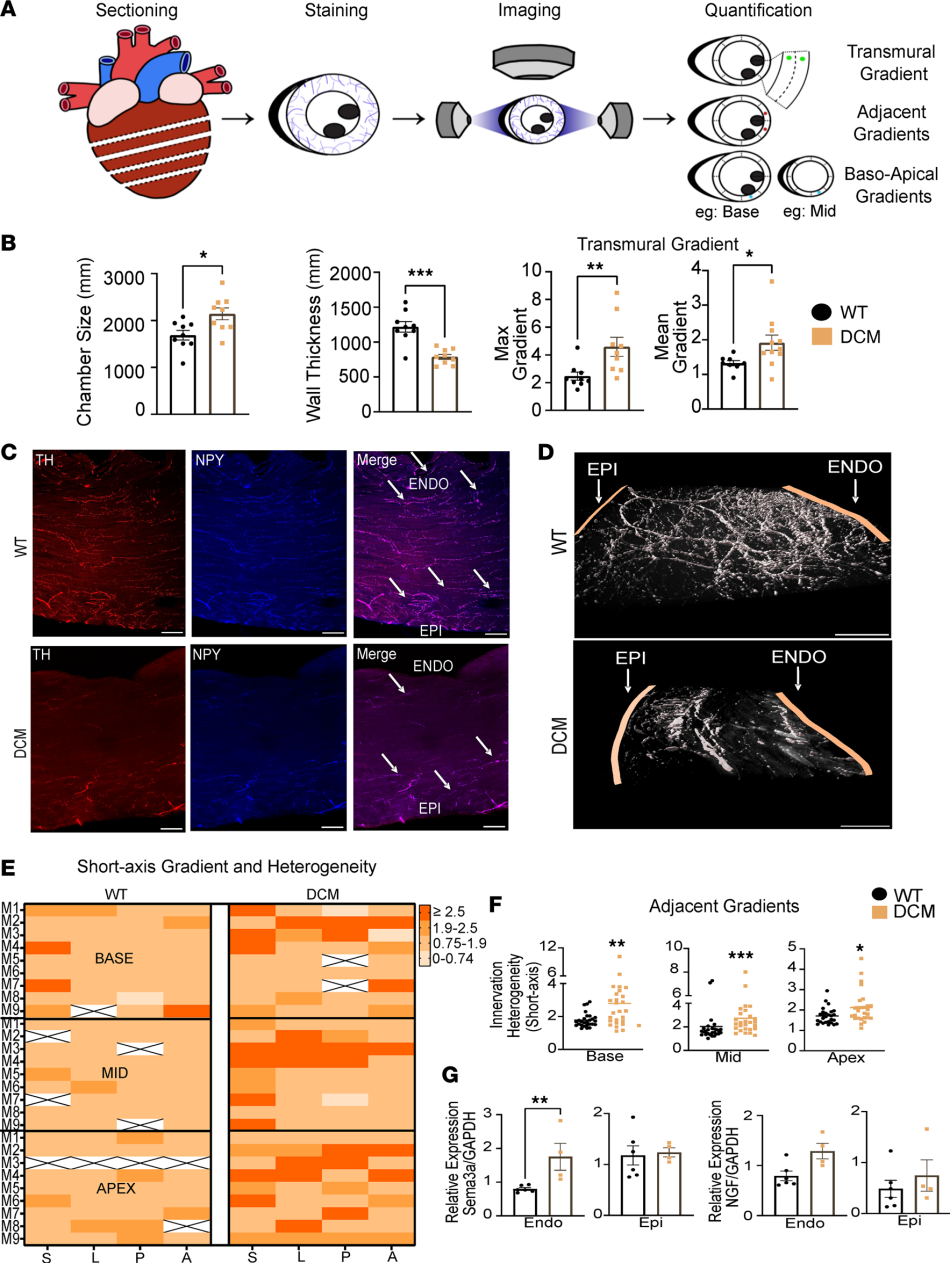
DCM model shows a sympathetic innervation gradient and heterogeneity in innervation in the short-axis orientation. (**A**) Schematic illustrating calculation of innervation gradients. (**B**) Comparisons of left ventricule (LV) internal diameter (*n* = 9 control, *n* = 9 DCM, *P = 0.0135, Shapiro-Wilk test, Welch’s *t* test), LV wall thickness (*n* = 9 control, *n* = 9 DCM, ***P = 0.0003, Shapiro-Wilk test, Welch’s *t* test), maximum epicardial to endocardial innervation gradient (*n* = 9 for control, *n* = 9 for DCM, **P = 0.0019, Shapiro-Wilk test, Mann-Whitney test), and mean apical epicardial to endocardial gradient (*n* = 9 control, *n* = 11 DCM, *P = 0.0260, Shapiro-Wilk test, Welch’s *t* test). (**C**) Immunohistochemical staining of WT (top) and DCM (bottom) heart sections. White arrows highlight innervation levels in epicardium and endocardium (1 arrow = low innervation, 3 arrows = high innervation). Scale bars are 100 μm. (**D**) Imaris 3D reconstruction of sympathetic innervation of heart sections from WT (top) and DCM (bottom) mice. (**E**) Summary heatmap of innervation gradient by level (base, mid, apex) and region (S, septal; L, lateral; A, anterior; P, posterior). (**F**) Short-axis innervation heterogeneity in WT and DCM mouse sections across base (*n* = 9 for control, *n* = 9 DCM, **P = 0.005, Shapiro-Wilk test, Mann-Whitney test), mid (*n* = 9 control, *n* = 9 DCM, ***P = 0.0003, Shapiro-Wilk test, Mann-Whitney test), and apex (*n* = 9 control, *n* = 9 DCM, *P = 0.0146, Shapiro-Wilk test, Mann-Whitney test) regions. (**G**) Reverse transcription quantitative PCR (RT-qPCR) comparing relative mRNA levels of Sema3a between WT and DCM mice in endocardial (left, *n* = 6 control, *n* = 4 DCM, **P = 0.0092, 2-way ANOVA/Tukey’s multiple comparisons test) and epicardial regions (right, *n* = 6 control, *n* = 4 DCM). Relative mRNA levels of NGF in endocardial (left, *n* = 6 control, *n* = 4 DCM) and epicardial regions (right, *n* = 6 control, *n* = 4 DCM).

To test the hypothesis that loss of sympathetic innervation in DCM mice is spatially heterogeneous, we quantified sympathetic nerves (immunoreactivity to tyrosine hydroxylase and neuropeptide Y) across the entire left ventricle in short axis (anterior, septal, posterior, and lateral walls) and long axis (base, mid, and apex) (Figure 3A). As expected, DCM hearts exhibited LV chamber enlargement and wall thinning. However, compared with control hearts, DCM hearts exhibited greater denervation in the endocardium compared with the epicardium, resulting in transmural innervation gradients (Figure 3, B–D). As depicted in Figure 3E, increased transmural gradients were present at the base, mid, and apical levels of the LV. Interestingly, gradients were nonuniform across the anterior, septal, posterior, and lateral walls, such that sites with stark differences in transmural gradient were adjacent to each other (Figure 3F).

**Figure 4:**
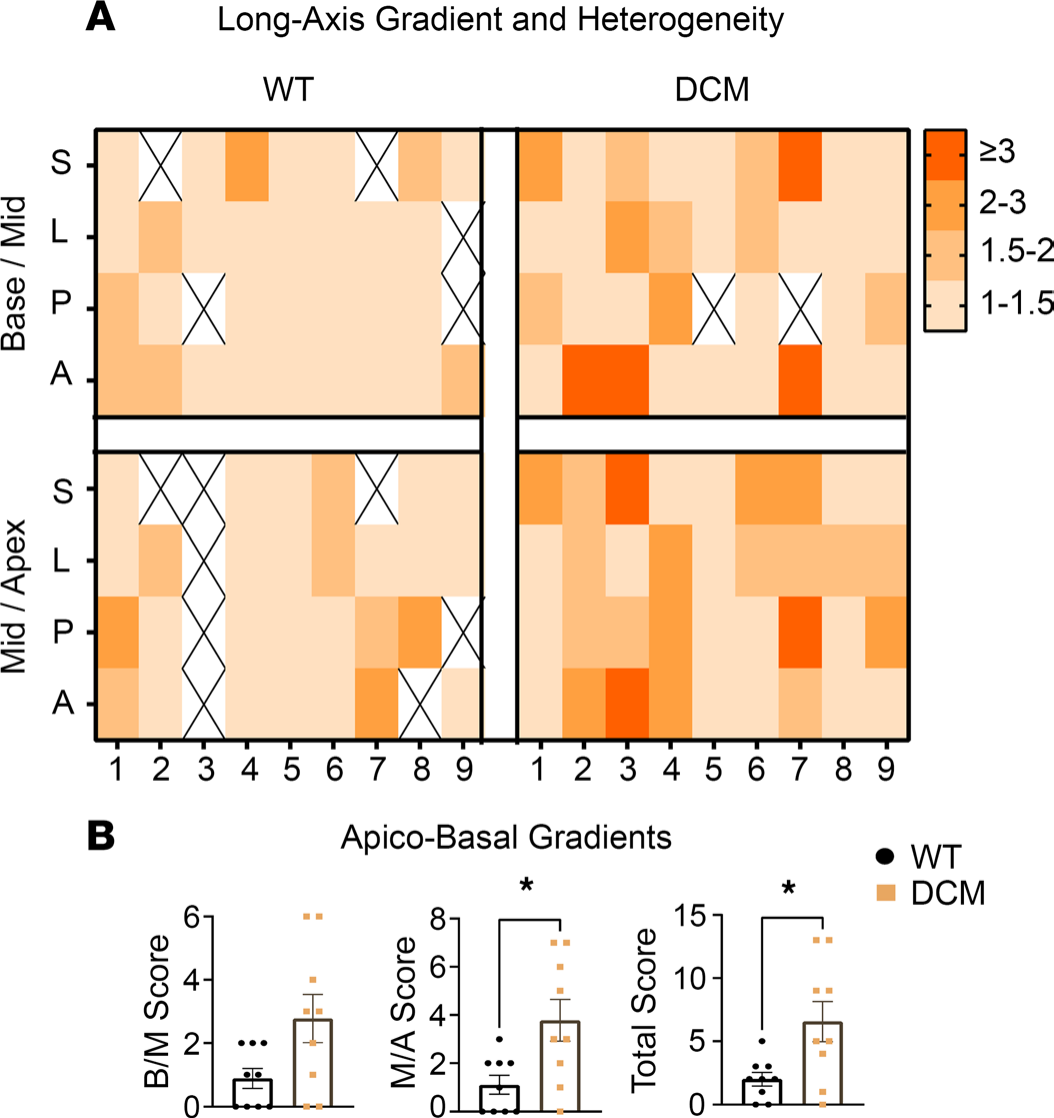
DCM model shows increased heterogeneity of sympathetic innervation gradients in the long-axis orientation. (**A**) Summary heatmap showing ratios of sympathetic innervation gradients by long-axis level comparison (Base/Mid, Mid/Apex) and region (S, septal; L, lateral; A, anterior; P, posterior). (**B**) Long-axis heterogeneity scores based on ratios of sympathetic innervation gradients between base/mid (B/M, *n* = 9 for control, *n* = 9 for DCM, *P* = 0.0644, Shapiro-Wilk test, Mann-Whitney test), mid/apex (M/A, *n* = 9 for control, *n* = 9 for DCM, *P = 0.0232, Shapiro-Wilk test, Mann-Whitney test), and total (scores of B/M and M/A combined, *n* = 9 for control, *n* = 9 for DCM, *P = 0.0216, Shapiro-Wilk test, Welch’s *t* test). Higher scores indicate increased heterogeneity in sympathetic innervation. Please refer to Figure 3A for visualization of the quantification method.

When quantified, DCM hearts showed great innervation heterogeneity (i.e., adjacent sites with disparate transmural gradients) compared with control animals (Figure 3F) at the basal, mid, and apical segments of the LV. We next sought to determine whether DCM hearts also exhibit innervation heterogeneity in the long axis (i.e. base-mid, and midapical segments). A ratio of transmural gradients in the long-axis orientation was found to be significantly higher when comparing both the base-mid and midapical segments across the anterior, septal, posterior, and lateral walls (Figure 4). To verify that these gradients were not isolated to the DCM Tg9 model, we examined a different transgenic mouse model of nonischemic cardiomyopathy and heart failure (HF). We compared mice with cardiomyocyte-specific overexpression of long-chain acyl-CoA synthetase 1 (*Acsl1*^+^), an established model of lipotoxicity, LV dysfunction, and HF (18). In this model, *Acsl1*^+^ hearts with DCM and HF showed greater denervation in endocardial segments compared with the epicardium and increased transmural sympathetic innervation gradients (Supplemental Figure 2, A–C).

**Figure 5:**
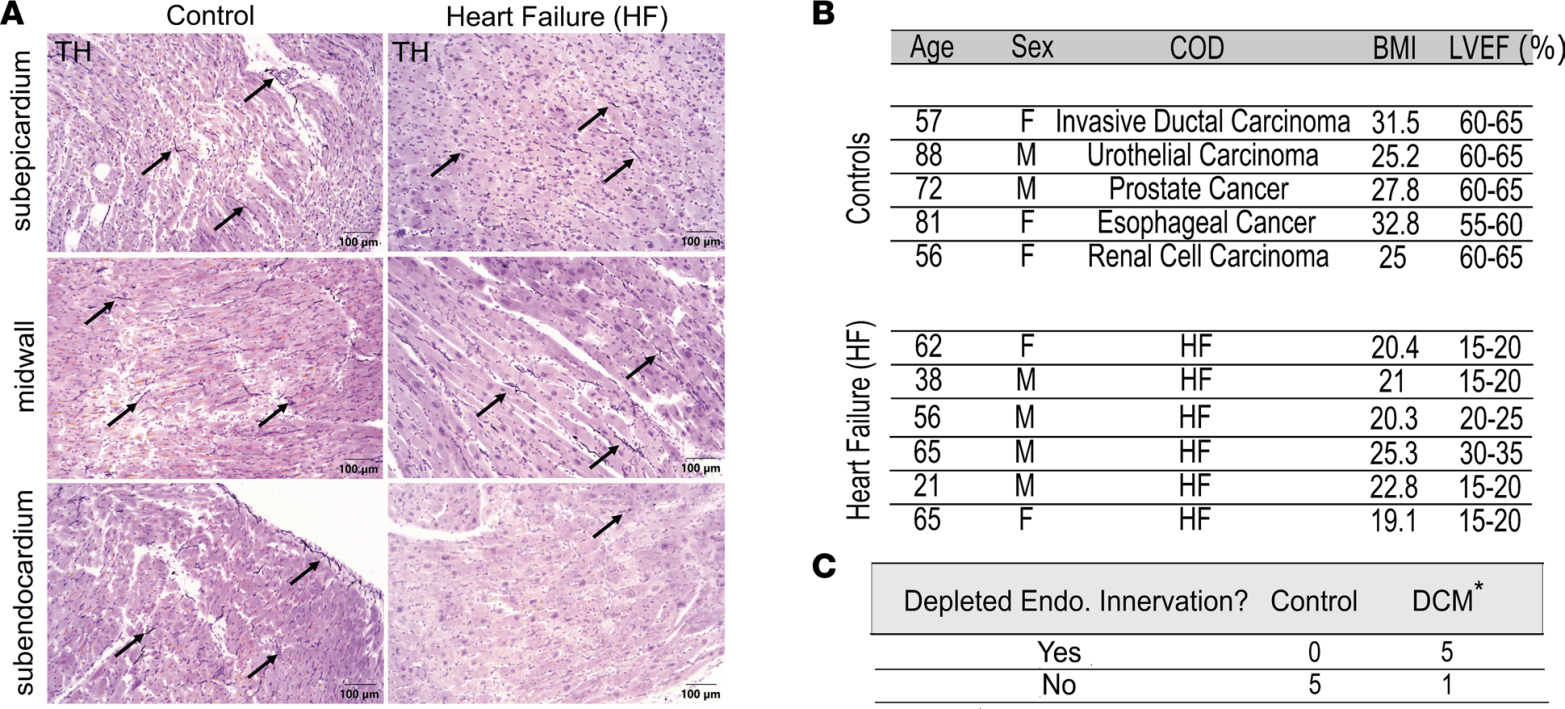
DCM model shows an increased gradient and heterogeneity in cardiac adrenergic innervation, as well as increased arrhythmogenesis. (**A**) Schematic of norepinephrine (NE) injections used to induce VAs in WT and DCM mice. (**B**) Arrhythmias (PVCs, premature ventricular contractions [**P = 0.0091]; couplets [*P = 0.0171]; NSVT, nonsustained ventricular tachycardia [*P = 0.0350]) from ECG recordings of control and DCM mice (*n* = 8 for control, *n* = 12 for DCM, χ^2^ test). (**C**) Examples of arrhythmias in DCM mice including individual PVCs, couplets, bigeminy, and NSVT (scale bar is 100 ms). (**D**) PVC count after NE injection (left, *n* = 8 for control, *n* = 12 for DCM, *P = 0.0308, Shapiro-Wilk test, Mann-Whitney test). Arrhythmogenicity index (right, *n* = 8 for control, *n* = 12 for DCM, *P = 0.0113, Shapiro-Wilk test, Welch’s *t* test) based on ECG recordings of WT and DCM mice. (**E**) Correlation between arrhythmogenicity index score and mean gradient of mice with a mean epicardial-to-endocardial sympathetic innervation gradient greater than 1.4 (*R*^2^ = 0.56, simple linear regression).

To verify the presence of a transmural gradient in human cases of nonischemic HF, blinded analysis was conducted on human samples of healthy controls and nonischemic HF to examine sympathetic nerves with immunoreactivity to tyrosine hydroxylase (TH) (Figure 5, A and B). Greater denervation on the endocardium compared with the epicardium was visually identified in a greater number of nonischemic HF samples compared with healthy control samples (Figure 5C).

Given that denervation in human HF and canine and rodent models of HF have implicated altered expression of neurotrophin/neurorepellant in cardiomyocytes, we sought to determine whether there are transmural differences in the neurotrophin NGF and the neurorepellant Sema3a in failing hearts versus WT. Transmural sections rapidly frozen and separated into epicardial and endocardial segments demonstrated greater mRNA expression of Sema3a in the endocardium of DCM compared with control hearts, while epicardial levels of Sema3a and endocardial or epicardial levels of NGF were not significantly different between DCM and WT hearts (Figure 3G).

### DCM hearts exhibited frequent and complex VAs.

**Figure 6:**
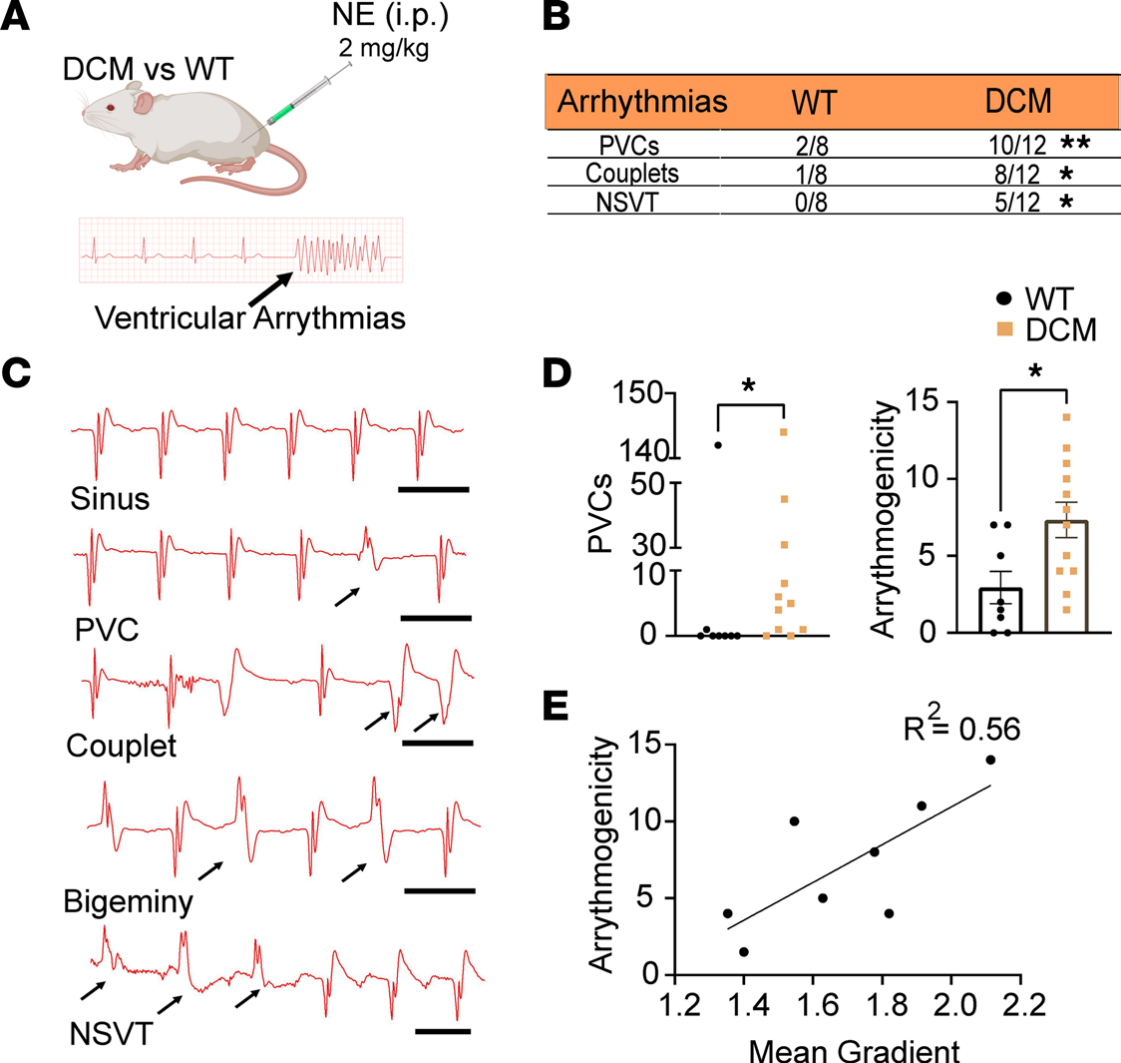
Human samples of DCM show a heightened sympathetic innervation gradient. (**A**) Digital imaging (original magnification, 20×) of IHC staining with TH on samples from patient controls (left, *n* = 5) and patients with HF (right, *n* = 6). Arrows indicate representative nerve fibers. (**B**) Patient demographic information (from left to right: age, sex [male or female], cause of death [COD], body mass index [BMI], and LVEF %). (**C**) Results from blind testing categorizing images as control or HF based on the presence of significantly depleted innervation in the endocardium (*P = 0.0152, *n* = 5 for control, *n* = 6 for HF, Fisher’s exact test).

To explore possible arrhythmic consequences of the increased innervation gradients, continuous ECG recordings were performed in DCM and control mice (Figure 6A) under light sedation (1%–2% inhaled isoflurane). Premature ventricular contractions (PVCs), couplets, and nonsustained ventricular tachycardia (NSVT) were present in a greater number of DCM mice compared with controls (Figure 6, B and C). Correspondingly, NE injection induced a significantly greater number of PVCs in DCM mice compared with controls (Figure 6D), which was also reflected in their higher arrhythmogenicity score (Figure 6D). Mean transmural innervation gradient and arrhythmogenicity score were positively correlated in DCM mice with mean gradients ranging from 1.4 to 2.2 (Figure 6E).

### Gradients in sympathetic innervation can generate spontaneous PVCs in HF computer simulations.

**Figure 7:**
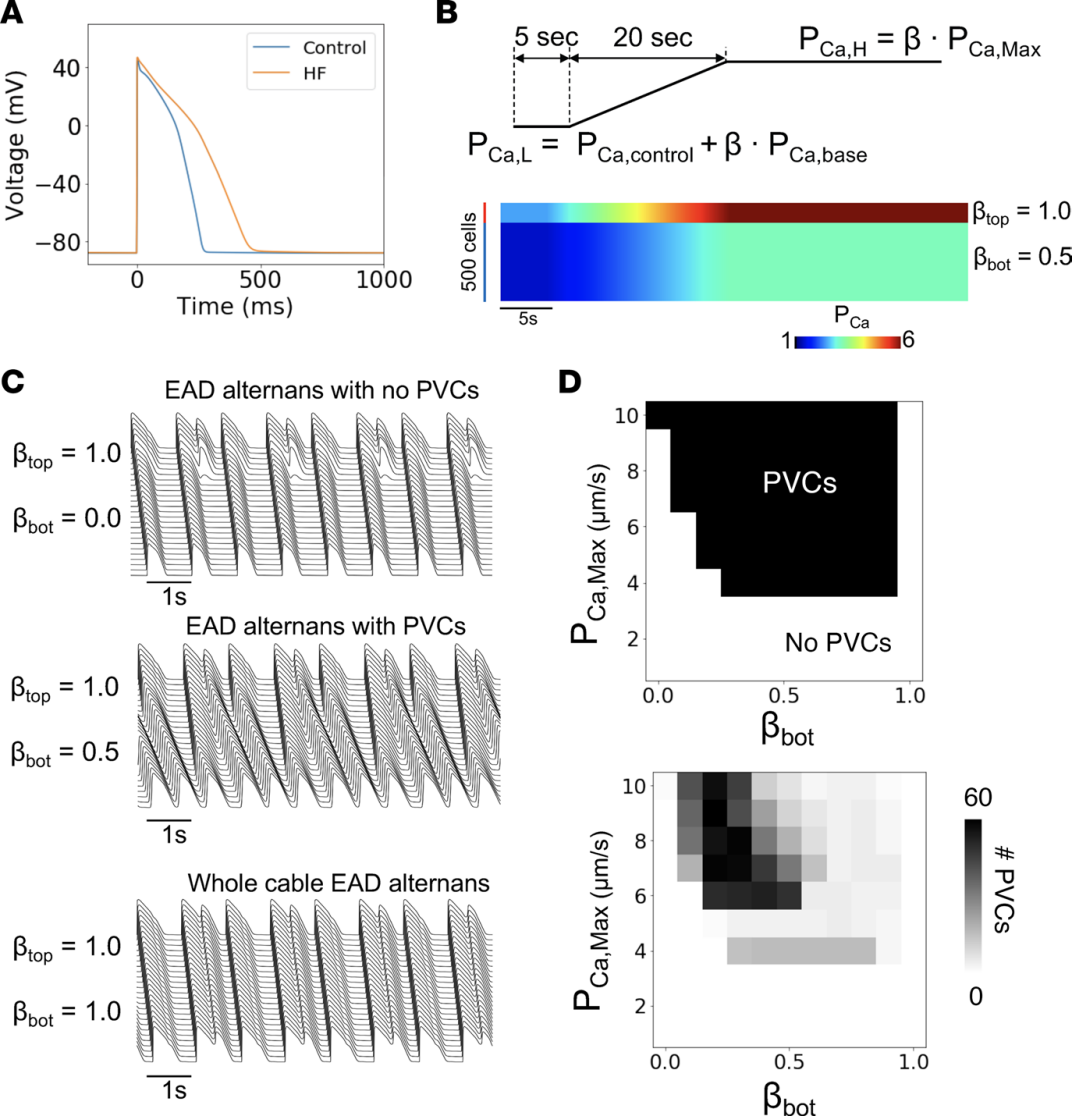
1D cable HF simulations with a gradient in β sympathetic innervation. (**A**) Voltage action potential of the control ORd model (blue) and the HF ORd model (orange). (**B**) Top: Schematic of L-type Ca current conductance (P_Ca_) ramp increase during a simulated β sympathetic surge. The increase in PCa is proportional to a β factor, which is the percentage of sympathetic innervation to that cell. Bottom: 500-cell cable with the top 100 cells having βtop = 1.0 and the bottom 400 cells having a variable βbot. Line scan shows an example with βbot = 0.5. (**C**) Three types of behaviors observed in HF simulation. Top: EAD alternans only in top part of the cable, middle: PVCs propagate out of the gradient region, bottom: whole-cable EAD alternans. (**D**) Top: Phase diagram of PVCs when varying P_Ca,Max_ vs. βbot. Bottom: Same phase diagram except with number of PVCs during the simulation.

To mechanistically investigate the arrhythmogenic effects of sympathetic innervation gradients, we performed computer modeling simulations of cardiac tissue (Supplemental Methods). First, as shown in Figure 7, A and B, we simulated a 1D cable consisting of 500 electrophysiological homogeneous endocardial cells. However, the top of the cable was fully innervated (β = 1.0) while the bottom of the cable varied from 0% to 100% innervated (β = 0.0–1.0), resulting in a gradient in L-type Ca current (I_CaL_) throughout the sympathetic surge ramp protocol. This resulted in several types of behaviors as shown in Figure 7C, which included early afterdepolarization (EAD) alternans with no PVCs (β_top_ = 1.0, β_bot_ = 0.0), PVCs that emerged and propagated from the gradient region (β_top_ = 1.0, β_bot_ = 0.5), and whole cable EAD alternans in the case of homogeneous innervation (β_top_ = β_bot_ = 1.0).

To investigate the conditions conducive for PVCs, we simultaneously varied both the maximum level of I_CaL_ conductance during a sympathetic surge (P_Ca,max_) as well as reduced the sympathetic innervation level for the β_bot_ while maintaining β_top_ = 1.0, which created a gradient of innervation in the cable. The resulting phase diagram shown in Figure 7D demonstrates several features. First, there is a minimum level of P_Ca,max_ (4 μm/s with these parameters) required for PVCs to occur. Second, once this minimum level is met, a larger denervation gradient (smaller β_bot_) generally results in more PVCs (see lower panel in Figure 7D). Finally, if the innervation level β_bot_ is reduced too low, PVCs are unable to form despite the presence of EADs (Figure 7C, top panel). Thus, larger gradients in sympathetic innervation appear to potentiate PVCs as long as there is still some minimum level of sympathetic activity in the reduced innervation region. The mechanism of repolarization gradients promoting PVCs has been demonstrated in our previous studies (19, 20), which showed that PVCs are caused by dynamical instability in tissue, promoted by the presence of EADs, repolarization gradient, and enhanced I_CaL_ conductance. In the case shown here, heterogeneous innervation and sympathetic surge result in EADs, increased repolarization gradient, and enhanced I_CaL_, which set the tissue condition for PVC genesis.

### Heterogeneity of sympathetic innervation gradients in HF promotes reentrant arrhythmias.

**Figure 8:**
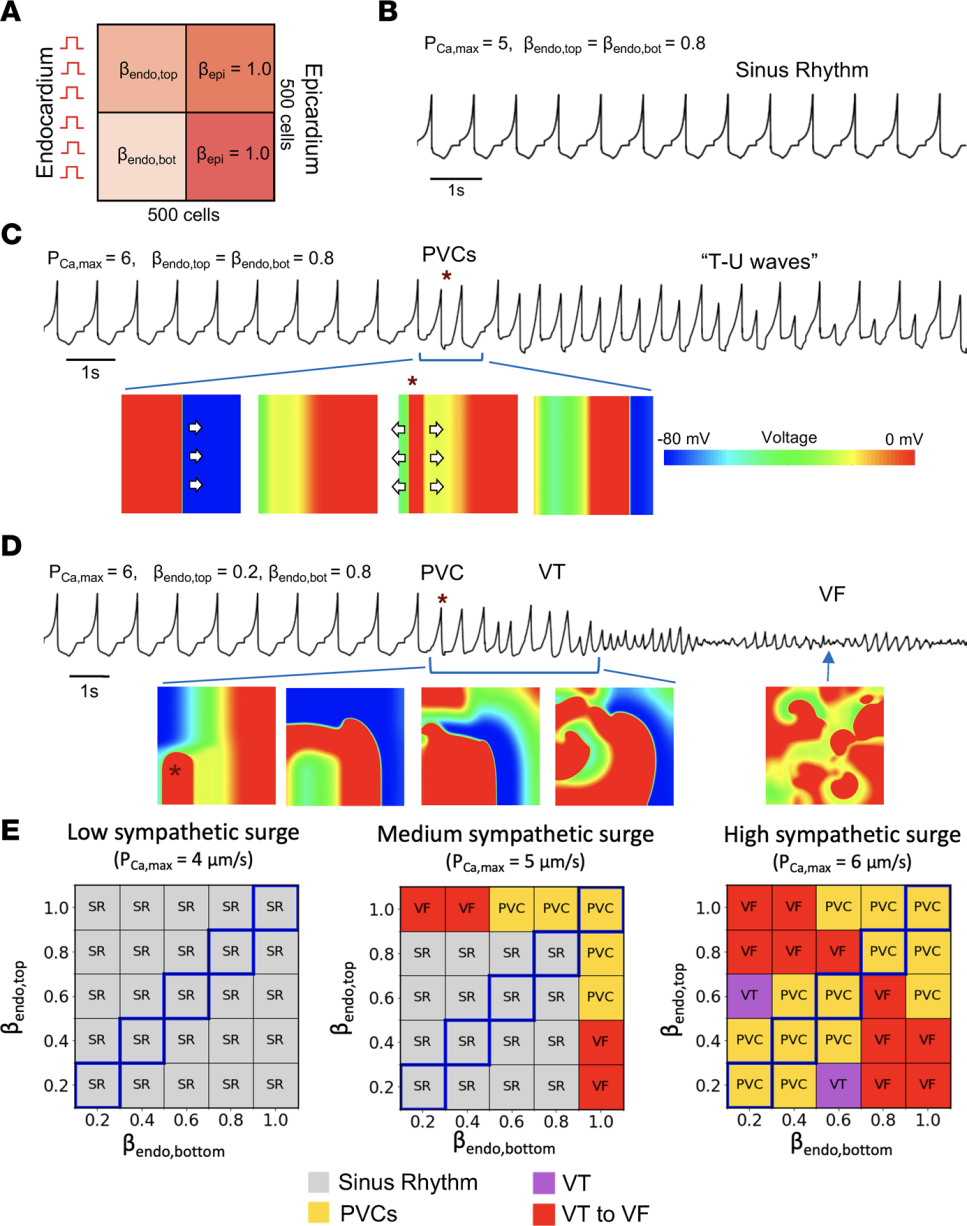
2D tissue HF simulations of cardiac tissue with heterogeneity in endocardial sympathetic innervation. (**A**) Schematic of 2D simulation setup. A 500 × 500 cell tissue is divided into 3 regions. The epicardial region is always fully innervated with β_epi_ = 1.0. The endocardial region has 2 subregions with varying β_endo,top_ and β_endo,bot_ values to simulate varying degrees of denervation. P_Ca_ is ramped up as shown in **B** to simulate a sympathetic surge. (**B**) Pseudo-ECG where P_Ca,max_ = 5, β_endo,top_ = β_endo,bot_ = 0.8 with normal sinus rhythm. (**C**) Pseudo-ECG and voltage snapshots where P_Ca,max_ = 6, β_endo,top_ = β_endo,bot_ = 0.8, resulting in PVCs (example marked with *) and T-wave repolarization abnormalities but no reentrant arrhythmias. (**D**) Same where P_Ca,max_ = 6, β_endo,top_ = 0.2, and β_endo,bot_ = 0.8, resulting in PVCs, ventricular tachycardia (VT) reentrant arrhythmia, and eventually ventricular fibrillation (VF). (**E**) Phase diagram of observed behaviors for different β_endo,top_ versus β_endo,bot_ values at P_Ca,max_ = 4, 5, 6, respectively. The blue bolded diagonal corresponds to cases of homogeneous innervation gradients.

To further investigate the effects of differing endocardial-epicardial innervation gradients in HF as seen in our experimental results, we next performed 2D cardiac tissue simulations incorporating a simplified endocardial-epicardial geometry. The simulation setup is shown in Figure 8A, with a 500 × 500 cell tissue divided from left to right into endocardial and epicardial regions and from top to bottom with different sympathetic innervation gradients. For simplicity, β_epi_ = 1.0 for both the top and bottom, while β_endo,top_ and β_endo,bot_ was varied from 0.2 to 1.0. The endocardial and epicardial regions have the appropriate HF electrophysiological parameters as detailed in Methods.

These 2D simulations exhibited several behaviors depending on the denervation pattern. Figure 8, B–D, show the pseudo-ECGs and tissue voltage snapshots of several parameter combinations. In Figure 8B, simulating a normal heart with normal cellular electrophysiologic function and without heterogeneity in the epicardial-endocardial innervation gradients (β_endo,top_ = β_endo,bot_ = 0.8), we observed only normal sinus rhythm at a P_Ca,max_ of 5 μm/s. However, in Figure 8C once we increased P_Ca,max_ to 6 μm/s (still in the homogeneous gradient case with β_endo,top_ = β_endo,bot_ = 0.8), we now observed PVCs and T-wave repolarization abnormalities (the pseudo-ECG equivalent of T-U waves, caused by an EAD occurring on the wave back without PVC propagation). Finally, in Figure 8D with heterogeneity in innervation gradients present (P_Ca,max_ = 6 μm/s, β_endo,top_ = 1.0, β_endo,bot_ = 0.2), we now saw a clear VT to VF reentrant arrhythmia. The asymmetry in innervation gradients allowed for a PVC to originate from one of the endo-epi gradients but not the other, leading to a reentrant arrhythmia forming, which eventually underwent wave break into VF.

As in the previous cable simulations, we simultaneously varied the P_Ca,max_ and the sympathetic innervation level to elicit different behaviors. This time, however, as we had 2 regions of different innervation in the endocardium, we had to vary both β_endo,top_ and β_endo,bot_, resulting in a 3D phase diagram. Figure 8E shows several cuts of this arrhythmia phase diagram with β_endo,top_ plotted against β_endo,bot_ for 3 values of P_Ca,max_ = 4, 5, 6 μm/s, representing a low, medium, or high sympathetic surge, respectively. The identity diagonal along the plot represents a homogeneous epi-endo gradient across the tissue. The off-diagonal elements represent varying degrees of heterogeneity of epi-endo gradients between the top and bottom of the tissue, as defined by β_endo,top_ and β_endo,bot_. With P_Ca,max_ = 4 μm/s, only sinus rhythm was observed at all combinations of gradients. As the sympathetic surge was stronger with P_Ca,max_ = 5 μm/s, we began to see PVCs or VF appear with certain combinations of endocardial innervation heterogeneity. Note that the severe arrhythmias only manifested with large heterogeneity of gradients, the top left and bottom right corners of the plot. At an even stronger sympathetic surge with P_Ca,max_ = 6 μm/s, all combinations of gradients exhibited some arrhythmia, but the diagonal representing homogeneous gradients exhibited only benign PVCs, while the large-heterogeneity off-diagonal regions again exhibited more severe VT and VF arrhythmias.

**Figure 9:**
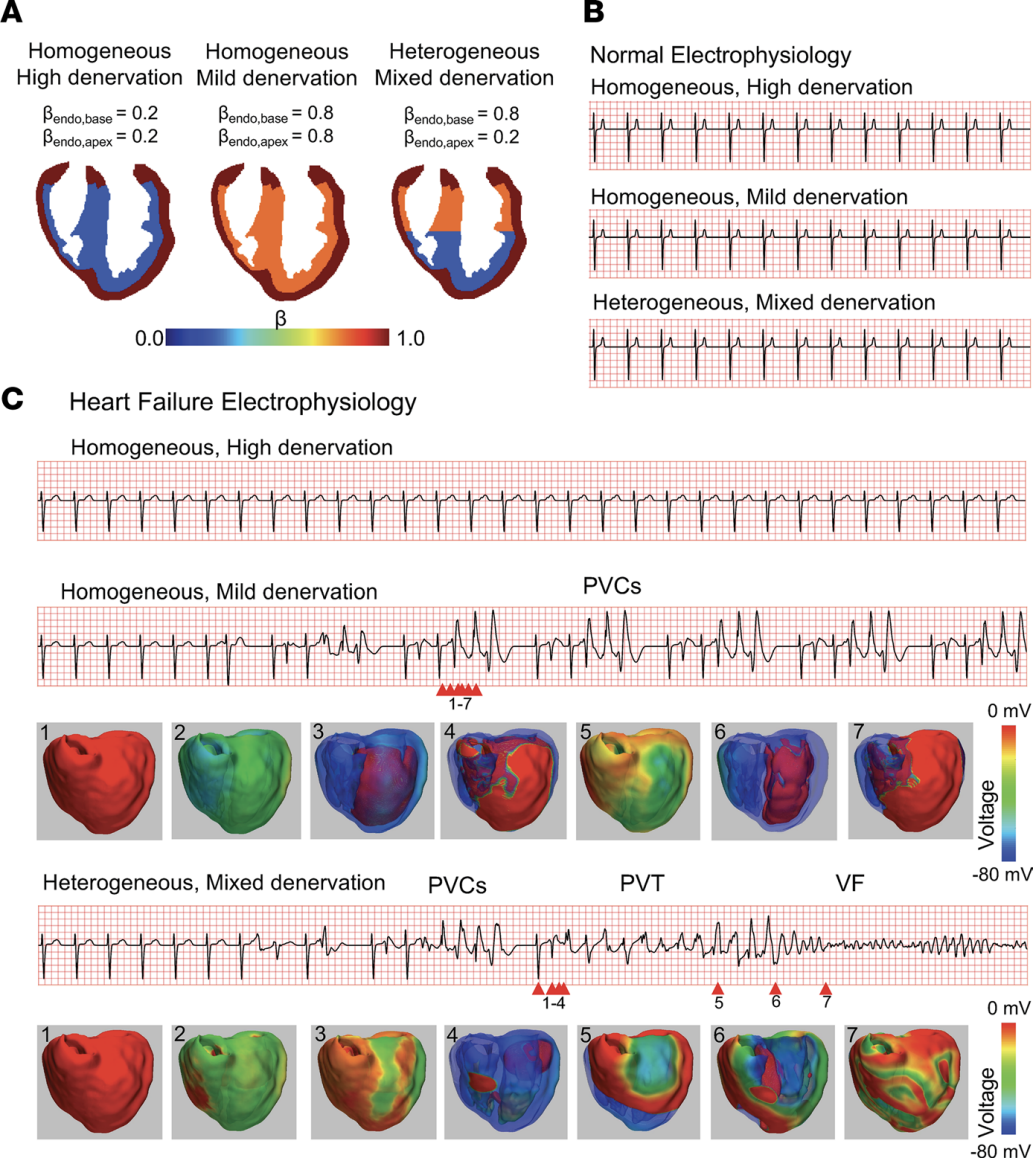
Whole-ventricle simulations with varying heterogeneity in endocardial sympathetic innervation. (**A**) Schematic of the epicardial and 2 endocardial regions that have varying degrees of sympathetic innervation. βepi = 1.0 for all cases. βendo,base and βendo,apex vary to form a gradient in sympathetic response. PCa is ramped up as shown in **B** to simulate a sympathetic surge. (**B**) ECGs under normal (non-HF) electrophysiology for 3 cases of different endocardial denervation patterns during a sympathetic surge. Stable sinus rhythm without arrhythmias is maintained. (**C**) ECGs under HF electrophysiology for the same 3 cases of different endocardial denervation patterns. Top ECG: High denervation but no gradient (βendo,base = 0.2, βendo,base = 0.2) results in sinus rhythm. Middle ECG: Low denervation but no gradient (βendo,base = 0.8, βendo,base = 0.8) results in repeating PVCs. Voltage snapshots of the whole ventricle are shown below, numbered to correspond to the arrows on the ECG. Bottom ECG: Mixed denervation with a strong gradient (βendo,base = 0.8, βendo,base = 0.2) results in a polymorphic VT to VF arrhythmia. Voltage snapshots shown below, numbered corresponding to the arrows.

Finally, to verify these results with a more realistic physiological geometry, we then performed simulations using a 3D anatomical ventricle geometry. The simulation setup is shown in Figure 9A, similar to the 2D simulations. An epi-endo innervation gradient was created again with β_epi_ = 1.0 and β_endo,base_ and β_endo,apex_ varying between the base and apex regions of the endocardium. Three different scenarios were simulated: homogeneous high denervation (β_endo,base_ = 0.2, β_endo,base_ = 0.2), homogeneous mild denervation (β_endo,base_ = 0.8, β_endo,base_ = 0.8), and heterogeneous mixed denervation (β_endo,base_ = 0.8, β_endo,base_ = 0.2). Figure 9B shows computed ECGs for the 3 scenarios with normal non-HF electrophysiology. In all 3 scenarios, we only saw sinus rhythm with no arrhythmias.

Figure 9C shows the 3 scenarios but now with HF electrophysiology. With homogeneous high denervation, we saw QT prolongation with sinus rhythm. With homogeneous mild denervation, we saw a repeating pattern of PVCs. As the apex and base are homogeneous in terms of innervation gradients, the PVC emerged evenly from the endo-epi boundary yet seemed to favor the LV in this example. With heterogeneous mixed denervation, we saw several PVCs, which then developed into a polymorphic VT, eventually degenerating into VF. In this case, we saw the PVC emerge from the endo-epi boundary mainly in the base region where β_endo,base_ = 0.8, though again favoring the LV (see voltage snapshot 4). The apex region this time did not excite ectopically because of the heterogeneity of denervation where β_endo,apex_ = 0.2, and thus the PVC was able to propagate asymmetrically from base to apex. A focal polymorphic VT developed, and this asymmetry eventually resulted in a competing focus developing, causing wave break, which degenerated into VF.

## Discussion

This study demonstrates that 1) heterogeneity in sympathetic innervation is characteristic of DCM in mice and humans and 2) this heterogeneity seemingly increases susceptibility to VAs. We show that DCM mice experience *functional* sympathetic ventricular denervation, as demonstrated by [^11^C]-mHED PET-CT, which was mirrored by an increased epicardial-to-endocardial sympathetic innervation gradient in the left ventricular myocardium on IHC. Moreover, we directly translate these findings to the clinical setting, as similar innervation patterns were observed in human HF tissue. Last, we correlate these changes in innervation gradients to increased arrhythmogenesis through in vivo ECG recordings with a NE challenge and through computer models that assess the arrhythmogenic consequences of the observed innervation gradients.

To our knowledge, this is the first study characterizing spatial dispersion of sympathetic innervation in HF and directly correlating these patterns to increased arrhythmogenesis. This evidence suggests that the DCM-induced changes in sympathetic innervation patterns play a fundamental role in promoting pro-arrhythmic conditions.

### Cardiac autonomic denervation in HF.

Prior animal and human studies have demonstrated that HF is associated with cardiac autonomic denervation. For example, Machado et al. showed that patients with DCM associated with Chagas disease experienced cardiac sympathetic denervation (21). Similarly, our murine model of DCM also displayed functional denervation on [^11^C]-mHED PET-CT data, which was additionally replicated in another murine model of HF in which HF was induced by an overexpression of Acsl1.

Cardiac sympathetic innervation is established during embryonic development, when sympathetic nerves reach the heart and traverse the myocardial wall from the epicardium to the endocardium (22, 23). Correspondingly, healthy mice exhibit an innervation gradient across their ventricular walls, with the highest density of nerves in the epicardium (24). Interestingly, spatial heterogeneity in cardiac sympathetic denervation, possibly resulting from NGF imbalance, has been linked to ventricular arrhythmogenesis (4, 25). Sema3a and Ngf could be fundamental in creating such heterogeneous innervation patterns. For example, Ieda et al. showed that altering murine cardiac exposure to Sema3a altered the transmural sympathetic gradient and increased the susceptibility to VAs (24). Moreover, heterogeneous sympathetic innervation was observed in canines susceptible to spontaneous VAs and sudden cardiac death (26).

At the tissue level in our study, cholinergic innervation was localized primarily to the epicardium as shown in IHC staining for vesicular acetylcholine transporter (VAChT) and did not differ between DCM mice and controls (Supplemental Figure 3). In contrast, the naturally present epicardial-to-endocardial sympathetic innervation gradient was exacerbated in mouse HF models and in human samples of nonischemic HF compared with controls. Differences in sympathetic innervation patterns of mice were associated with differences in Sema3a expression. Sema3a is a semaphorin implicated in the repulsion and guidance of axons (27, 28). Our study shows that this semaphorin was expressed at a significantly higher level in the endocardium of DCM mice compared with controls (Figure 3G), which could explain the heightened epicardial-to-endocardial gradient in this HF model (29, 30). Moreover, short- and long-axis heterogeneity of neighboring gradients was also higher in the DCM model in our study, which corresponds to findings in human tissue associating higher levels of catecholamine variability across 8 regions of the left ventricle to CHF (31). Importantly, the role of P75NTR receptor in modulating the sensitivity of sympathetic terminals to Sema3a is also a consideration. The phenotype identified in a prior study (32) investigating *P75NTR*-null mice bears resemblance to ours and clearly indicates a possible role for NGF/P75NTR signaling in DCM.

### Spatial dispersion of sympathetic innervation and ventricular arrhythmogenesis.

Additionally, Chen et al. recently showed that in patients with nonischemic cardiomyopathies, global sympathetic denervation correlates with the extent of ventricular fibrosis reflected in mean unipolar voltage, which is related to an increased risk for arrhythmogenesis (33). Prior studies have shown that denervated, but still viable, cardiac tissue is likely to be more sensitive to circulating catecholamines, which can promote arrhythmogenesis (34). Similarly, the DCM mice included in the current study showed an increased prevalence of VAs under baseline and after NE injection. This susceptibility was associated with a mean innervation gradient between 1.4 and 2.2.

In this study, we used computer modeling to explore the mechanistic arrhythmia implications of our experimental results, namely how HF can result in heterogeneous denervation of sympathetic fibers. Our simulations showed that gradients in sympathetic denervation can generate PVCs during a sympathetic surge through our previously described R-from-T mechanism. However, some minimal threshold of sympathetic innervation was still required for PVC formation. This is consistent with our previous studies (19, 20, 35, 36), where we demonstrated that excess I_CaL_ was required for PVC formation. If too much denervation is present, there is no longer enough sympathetic induced activation of I_CaL_ for the PVC to occur. Therefore, while in general larger innervation gradients were more conducive to PVCs, there exists an intermediate range where PVCs can form.

In addition to isolated innervation gradients, we investigated the effects of gradient heterogeneity, i.e., neighboring regions with different epicardial-endocardial innervation gradients as seen in our experimental results. Both our 2D and 3D anatomical ventricle simulations demonstrated that while PVCs could form with homogeneous innervation gradients, heterogeneity in innervation gradients was required for more severe reentrant and complex arrhythmias such as VT and VF. This arises from the fact that since the main gradient is from the endocardium to the epicardium, any PVC that forms from that gradient will be largely planar, traveling a short distance transmurally until hitting the surface boundary. This geometry of ectopic propagation will always be relatively benign, as the entire PVC will be extinguished quickly without any symmetry breaking. Conversely, with heterogeneous innervation gradients, the spontaneous PVC is now able to propagate longitudinally with much more room and asymmetry for reentry and/or wave break to occur.

Crucially, as these arrhythmias follow the R-from-T mechanism, both the sympathetic surge as well as QT prolongation must be present. Therefore, the electrophysiological changes of HF were a necessary component, along with the heterogeneous innervation gradients, for these arrhythmias to occur. Gradient heterogeneity in the setting of normal healthy electrophysiology was not able to produce arrhythmias.

The arrhythmia mechanisms demonstrated by computer modeling appear to be consistent with our experimental arrhythmia results as seen in Figure 6. Just as in the simulations, the DCM mice with increased epicardial-endocardial innervation gradients demonstrated more arrhythmias, mainly PVCs. Sustained VT or VF arrhythmias were rare in the mouse experiments, which may be due to the small size of the mouse heart, smaller than the tissue wavelength required for reentry (37). Regardless, one advantage of our combined experimental-computational approach is the ability to explore the arrhythmogenic effects across species boundaries from mice to humans. Using the general findings of heterogeneous sympathetic denervation from our in vivo mouse experiments, our computer simulations were performed using human models with human-sized tissue and ventricles, which were then large enough to form more complex arrhythmia patterns that are difficult in the small mouse heart.

These modeling results suggest that while innervation gradients from HF alone can produce PVCs, more severe arrhythmias may require additional conditions including a heterogeneity of innervation gradients. This may play a role in how early mortality from less severe HF (NYHA II-III) is primarily from sudden cardiac death (SCD), while mortality in severe HF (NYHA IV) is primarily from CHF (38), though this may be related to competing causes of death. If the initial denervation remodeling is patchy, the gradient heterogeneity may be more conducive to severe arrhythmias with higher risk of SCD. As the disease progresses and further remodeling occurs, the denervation may become more global, resulting in a more homogeneous gradient distribution, even though the individual gradients may be larger. This could have effects on the heart’s inotropy but may lessen the impact of arrhythmias as the global denervation may favor PVCs instead of more complex or sustained arrhythmias. This point would be worth exploring in future studies. The findings of the present study suggest that future therapeutic approaches that restore innervation gradients may reduce arrhythmias in patients with nonischemic cardiomyopathy, adding to the armamentarium of approaches to prevent sudden death in humans.

### Limitations.

This study was partially performed in a murine model of DCM. Murine pathophysiology and electrophysiology might incompletely translate to humans. However, sympathetic innervation gradients were also observed in human HF tissue. Further, using a computational model of human HF, the arrhythmogenic effects of this gradient were also shown. It should also be noted that this study demonstrates an association and not a causal link between myocardial sympathetic innervation gradients and arrhythmogenesis.

For the computer simulations, limitations include that only one geometry of anatomical ventricle was simulated. Patient-specific anatomical differences may influence the patterns of arrhythmogenesis from these mechanisms. For simplicity and mechanistic clarity, the heterogeneity of innervation gradients in the anatomical ventricle simulations was also only modeled as 2 large regions, base versus apex. Our experimental data show that there can be patchy distributions of innervation gradient heterogeneity, which may influence the initiation of complex arrhythmias. The adrenergic surge was modeled by ramping up I_CaL_ without any other effects, focusing on the initial phase of sympathetic simulation. While this should be sufficient for arrhythmia initiation, the next dominant current that β-adrenergic stimulation affects is slow inward rectifying potassium current, which has a slower time course than I_CaL_ and may play a role in arrhythmia termination (39). The sympathetic denervation was modeled as a percentage reduction in I_CaL_ ramp for simplicity, but the true effect may be nonlinear. The arrhythmia provocation studies relied on norepinephrine injections, which likely reflect the arrhythmogenic effects of adrenergic receptor sensitivity orchestrated by the nerve gradients, rather than directly from the altered nerves. Finally, HF throughout the disease course may cause other remodeling effects in addition to the electrophysiological changes and sympathetic denervation in our models.

### Conclusion.

DCM is characterized by the development of sympathetic innervation gradients in the left ventricular myocardium. This heterogeneity promotes ventricular arrhythmogenesis and could thus be an interesting target for future antiarrhythmic strategies.

## Methods

See supplemental materials for Supplemental Methods.

### Nonischemic mouse models of HF.

Details of the Tg9 DCM and Acsl1^+^ model (40) have been published previously. Both mice were obtained from laboratory stocks at UCLA and Washington University in St. Louis. This study included a total of 105 mice of both sexes aged 6–12 weeks. The number of animals used in each experiment is provided in each figure legend.

### Transthoracic echocardiography.

Echocardiography was performed on mice using a VisualSonics Vevo2100 system. EDD and LVEF (%) were assessed to assess left ventricular size and function.

### In vivo functional sympathetic innervation assessed by [^11^C]-mHED uptake.

See Supplemental Methods for synthesis of [^11^C]-mHED. Functional imaging of sympathetic innervation was assessed using PET-CT following [^11^C]-mHED injection. Mice were anesthetized with isoflurane mixed in oxygen (2%–3% induction, 1.5%–2% maintenance), secured to an imaging chamber on the Inveon PET scanner (Siemens Medical Solutions) or G8 PET/CT Scanner (PerkinElmer), and catheterized via tail vein. A 60-minute list-mode PET acquisition was started followed immediately by a bolus injection of [^11^C]-mHED via the catheter (specific activities ranged 94–110 Ci/mmol at injection, with mHED concentrations ranging 4–6 μg/mL). Mice imaged with the Inveon PET scanner underwent CT imaging on the CrumpCAT microCT (UCLA) for coregistration (Exitron 12000 was used as CT contrast). PET data were plotted as histograms into 8 × 5-minute frames (starting at 2.5 minutes after injection), corrected for isotope decay and detector normalization (and photon attenuation for the G8 scanner), reconstructed using OSEM-MAP or MLEM for G8 scanner, and normalized to %ID/g. Images were quantified by drawing regions of interest (14 mm^3^) on select tissues using Amide software (SourceForge).

### Arrhythmia induction.

Ventricular arrhythmogenesis was quantified in mice anesthetized under inhaled isoflurane (1%–2%). Recordings were made through subcutaneous needle electrodes in the right forearm and left hind leg to acquire lead II electrocardiograms (Grass RPS 107 Regulated Power Supply, Grass Instrument Co.; Grass P511 AC Amplifier, Natus Neurology). LabChart 8 was used to analyze ECG recordings for arrhythmias (AD Instruments). In short, 5-minute baseline recordings were obtained, after which NE (2 mg/kg) was injected intraperitoneally. After injection, ECG recordings were continued for over 30 minutes, or until death.

### Arrhythmogenicity index.

An arrhythmogenicity index was created to denote the severity of arrhythmogenesis based on ECG recordings from in vivo studies in mice, as has been done before in pigs (41). Arrhythmogenesis was scored based on time of induction and number of appearances (Supplemental Methods).

### Tissue preparation and imaging.

All IHC studies were performed on hearts following perfusion fixation. Briefly, following cervical dislocation, heparinized saline (50 U/mL) was injected into the left ventricular apex, followed by 4% paraformaldehyde (PFA) for 5 minutes. The heart was isolated and collected in a 0.01 M PBS solution, after which it was suspended in 4% PFA solution and stored at 4°C overnight.

Hearts were embedded in approximately 2% agarose solution in an upright position, and transmural sections of 100 μm thickness were obtained. Sections were stained for TH and NPY, or VAChT, using appropriate primary and fluorescent secondary antibodies (see Supplemental Methods for sources and catalog numbers). An LSM-880 confocal laser microscope was used to image the cardiac tissues (Zeiss). The following laser settings were used: 488 - PGP 9.5 (Green), 562 (Cy3) - TH (Red), 647 - NPY (Blue). Whole tissues were imaged at 10× original magnification and stitched together using tile scan. A representative ~20 μm thickness was designated to the *Z*-stack. All images were exported as.CZI files and converted to maximum intensity projections (MIPs) for subsequent quantitative analysis.

Deidentified left ventricular free wall samples from postmortem studies of patients with and without nonischemic HF were paraffin-embedded and sectioned at 4–6 μm. Samples were stained for TH using antigen retrieval ABC IHC (Supplemental Methods), and digital images (original magnification, 20×) were produced using an Olympus BX41 microscope aided by an Olympus DP74 digital camera and cellSens software (Olympus).

3D reconstruction of confocal microscopic images was performed using Imaris (Bitplane) to explore the morphology and distribution of sympathetic innervation. Intensity-based, 3D modeling was performed on heart sections stained with TH and NPY. The Surfaces tool was used on individual.CZI files of confocal images taken at original magnification, 20×, with *Z*-stacks of approximately 40 μm.

### RT-qPCR.

Mouse hearts were rapidly excised and placed on a metal tray on a slab of dry ice, cut in short axis, and rapidly separated into endo and epi regions under a large magnifying glass. Separated epicardial and endocardial tissues were then stored at –80°C. Tissues were homogenized with Trizol, and RNA was subsequently isolated and quantified through RT-qPCR using a NanoDrop device (Supplemental Methods).

### Quantification of cardiac fibrosis and sympathetic innervation.

Whole, transmural mouse heart sections were stained with Masson’s trichrome as described before (42) and were imaged with a Leica Biosystems Aperio AT Turbo digital pathology scanner. Images were analyzed on ImageJ software (NIH, USA). The color thresholding tool was used to measure total fibrosis area and total tissue area to calculate fibrosis (%).

Confocal images of mouse heart sections stained with TH and NPY were converted to MIP format. The left ventricle was divided into 8 segments separated by lines on the program Zen Black (Carl Zeiss), and the epicardium and endocardium were separated at the midpoint of the myocardium. The 16-bit, full-resolution images were analyzed on ImageJ software (NIH, USA). After binary conversion of the files, thresholding was used to calculate innervation area and tissue area of the epicardium and endocardium for all 8 segments. An epicardium-over-endocardium ratio was made by dividing the relative innervation area in the epicardium over the relative innervation area in the endocardium. Values quantifying the transmural gradient were further analyzed for heterogeneity by comparing a ratio of adjacent regions in the short-axis orientation and another ratio in the long-axis between identical regions in base-mid and midapex segments. Ratios from individual regions were scored to identify the severity of long-axis heterogeneity (Supplemental Methods).

### In silico studies of ventricular arrhythmogenesis.

Details of modeling studies are provided in Supplemental Methods. All computer simulations were performed on Tesla and GeForce GPUs (NVIDIA Corporation) with software written in the CUDA programming language.

### Statistics.

Statistical analysis was performed using GraphPad Prism 9.2.0. *P* < 0.05 was considered statistically significant. Data in all dot plots are shown as as mean ± SEM.

### Study approval.

All described studies were in accordance with the University of California Institutional Animal Care and Use Committee guidelines. All mice were housed in dedicated pathogen-free facilities at the UCLA. The UCLA Institutional Review Board approved human tissue studies.

### Data availability.

Supporting Data Values are available in the supplement.

## Author contributions

AHJD, MBL, JTL, RS, ZQ, and OAA designed research studies. AHD, LC, JC, MBL, MDP, EHS, CMW, MAO, JTL, MP, and RS conducted experiments/acquired data. AHD, LC, TJD, JC, MBL, MP, DBH, JTL, RS, ZQ, and OAA analyzed data. AJ, CVR, and PYJ provided reagents/samples. AHD, MBL, DBH, RS, and OAA drafted the manuscript. All authors approved the manuscript.

## Supplementary Material

Supplemental data

Supporting data values

## Version 1 10/10/2023

In-Press Preview

## Version 2 11/22/2023

Electronic publication
