# Supplementary material for: Heterogeneous cardiac sympathetic innervation gradients promote arrhythmogenesis in murine dilated cardiomyopathy
Source: JCI Insight. 2023 Nov 22;8(22):e157956. doi: 10.1172/jci.insight.157956 (PMC10721311; doi:10.1172/jci.insight.157956)
Supplement: Supplemental data [file jciinsight-8-157956-s230.pdf]

Supplemental Figures and Methods

HETEROGENEOUS CARDIAC SYMPATHETIC INNERVATION GRADIENTS PROMOTE ARRHYTHMOGENESIS IN MURINE DILATED CARDIOMYOPATHY

Al-Hassan J. Dajani[1], Michael B. Liu[1], Michael A. Olaopa[1], Lucian Cao[1], Carla Valenzuela-Ripoll[2], Timothy J. Davis[1], Megan D. Poston[3,4], Elizabeth H. Smith[3,4], Jaime Contreras[1], Marissa Pennino[1], Christopher M. Waldmann [5,6], Donald B. Hoover[3,4], Jason T. Lee[7,8], Patrick Y. Jay[9], Ali Javaheri[3], Roger Slavik[5], Zhilin Qu[1], & Olujimi A. Ajijola[1]

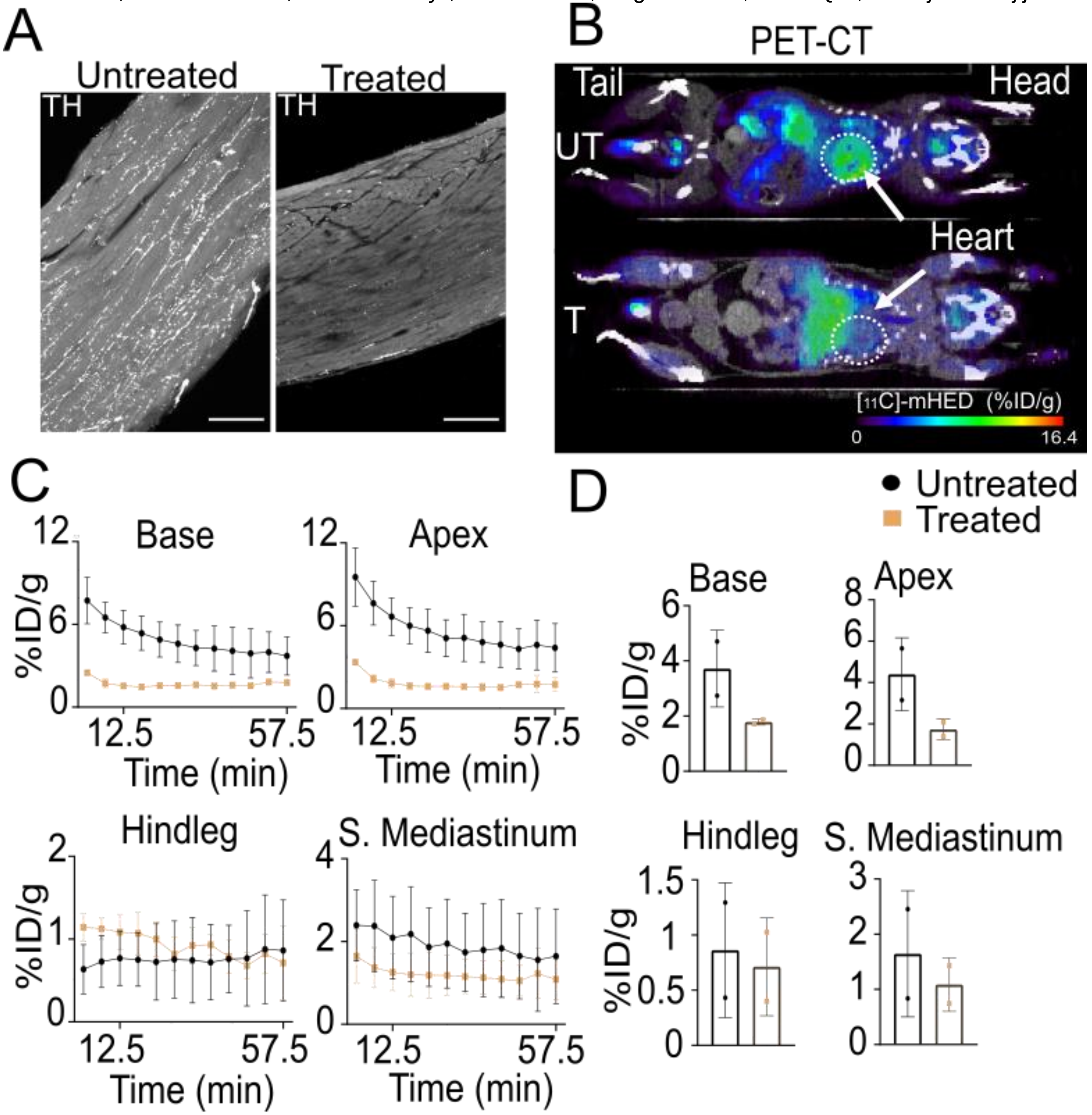

Supplemental Figure 1: Mice treated with 6-OHDA confirm sympathetic denervation. **A** Loss of sympathetic innervation in the myocardium confirmed through IHC staining for TH and NPY. Image scale bars are 100 µm. **B** [^{11}C]meta-Hydroxyephedrine positron emission tomography (PET-CT) images taken at the 60-minute time point scaled to percent injected dose per gram of tissue (% ID/g) of control mice (UT, Top) and mice treated with 6-OHDA (T, Bottom) (Head, Heart, and Tail labeled for orientation) **C** [^{11}C]meta-Hydroxyephedrine time-activity curves (0-60 minutes) showing uptake of [^{11}C]meta-Hydroxyephedrine in adrenergic nerve terminals of various tissues in control and treated mice. Analysis was conducted on mice pre- and post- treatment with 6-OHDA (n = 2 for control, n = 2 for DCM). **D** Quantification at 60 minutes to show uptake of [^{11}C]meta-Hydroxyephedrine in adrenergic nerve terminals of various tissues in control and treated mice: Cardiac Base, Cardiac Apex, Hindleg, Superior Mediastinum (n = 2 for control, n = 2 for DCM).

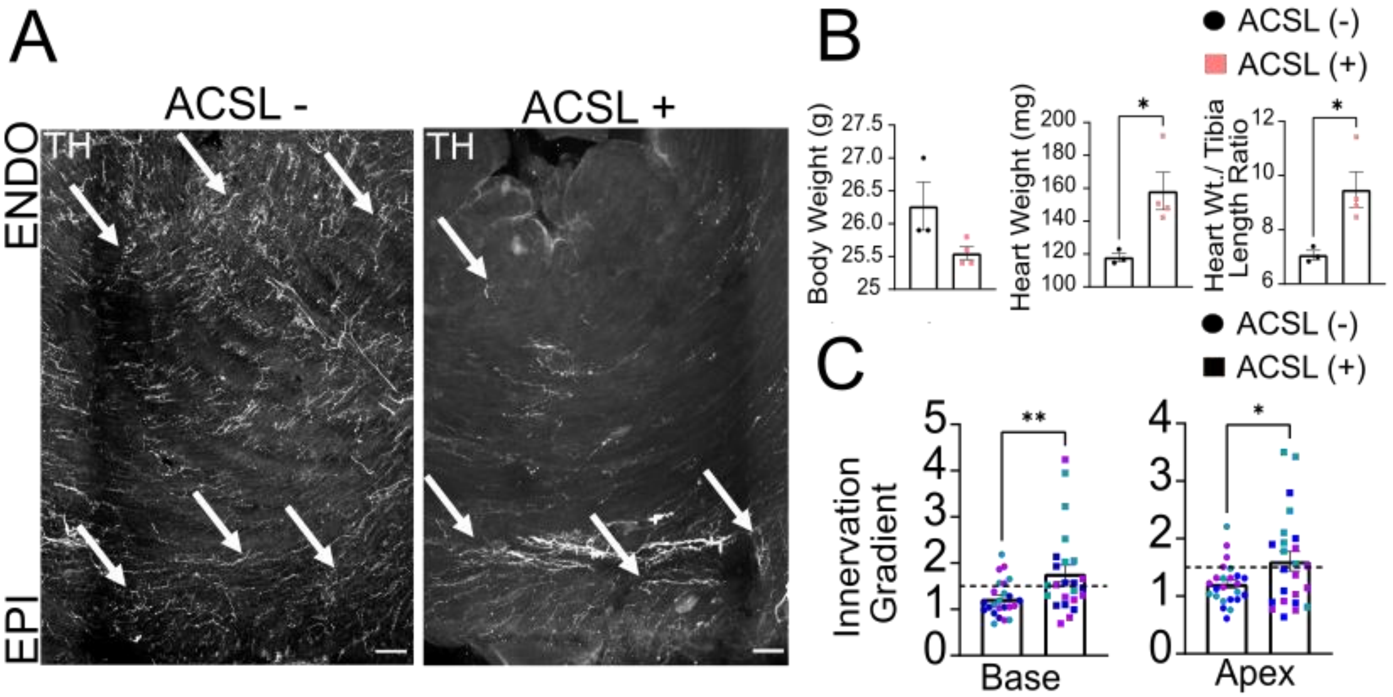

Supplemental Figure 2: ACSL Heart Failure model shows sympathetic innervation gradient.
A Confocal microscopy (10x) of IHC for TH in control (left) and ACSL + (right) heart sections (100 μm thickness) White arrows used to highlight innervation levels in Epicardium and Endocardium (1 arrow = "low innervation," 3 arrows = "high innervation"). Image scale bars are 100 μm. **B** Differences in body weight (p = 0.1836, Shapiro-Wilk test, Welch's t-test), heart weight (* p = 0.0355, Shapiro-Wilk test, Welch's t-test), and heart weight to tibia length ratio (* p = 0.0323, Shapiro-Wilk test, Welch's t-test) between control and ACSL+ mice (n = 3 for control, n = 4 for ACSL+). **C** Quantification of transmural sympathetic innervation gradient compared between control and ACSL+ mice at the base (n = 3 for control, n = 3 for ACSL+, **p = 0.0072, Kolmogorov-Smirnov test, Mann-Whitney test) and at the apex (n = 3 for control, n = 3 for ACSL+, *p = 0.0375, Kolmogorov-Smirnov test, Welch's t-test). The individual colors represent gradient values from all regions of left ventricle for individual mice. Image scale bars are 100 μm.

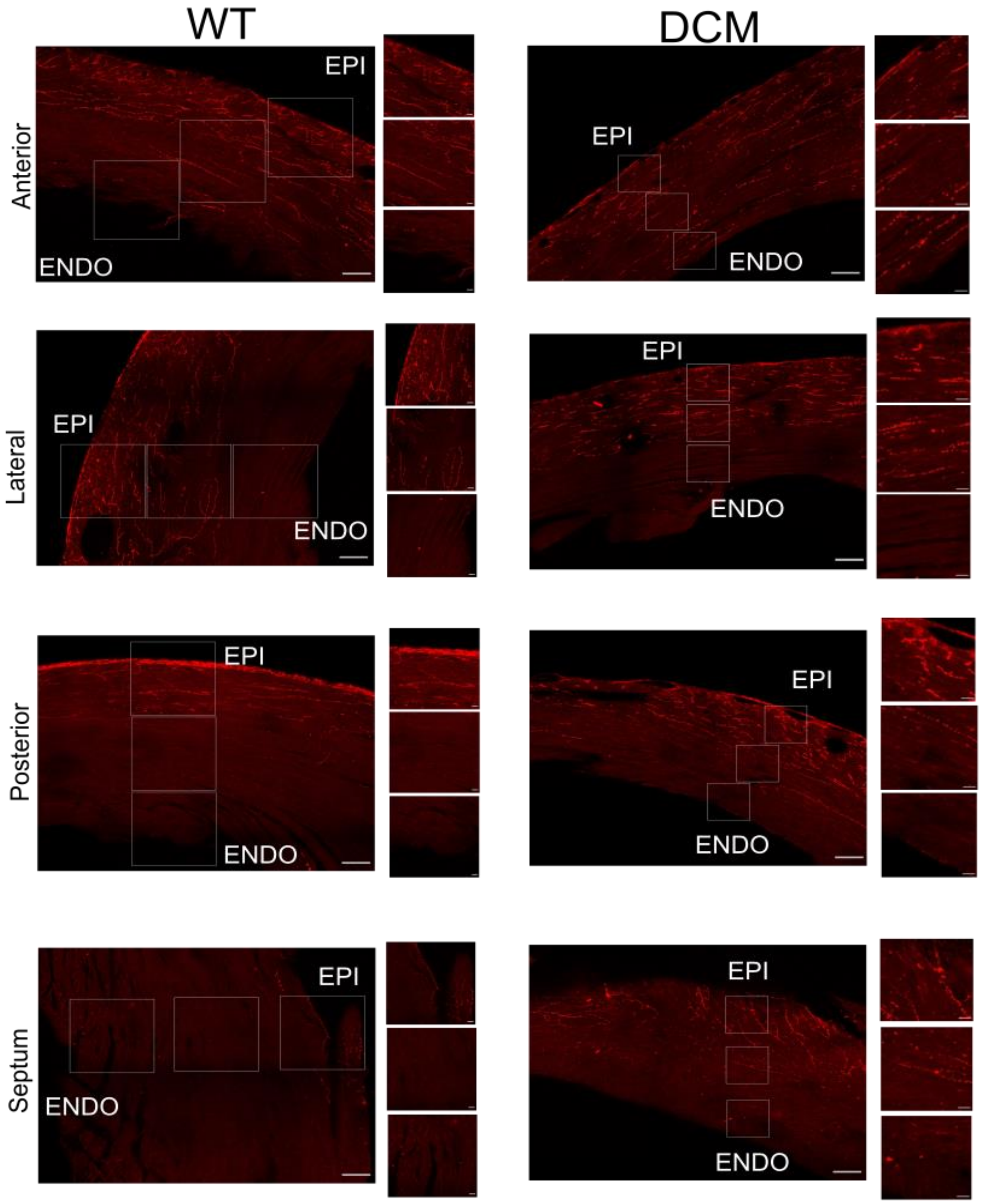

Supplemental Figure 3: Cholinergic staining of the ventricular myocardium is primarily restricted to the epicardium in DCM and control mice. Confocal microscopy (10x) of IHC with VAChT in control (left) and DCM mice (right) shows staining being localized in the epicardium across all four walls of the left cardiac ventricle (n = 4 for control, n = 4 for DCM). Image scale bars are 100 μm.

SUPPLEMENTAL METHODS

m-HED Synthesis Protocol

All commercially available reagents and materials were used as received. Dimethyl sulfoxide (DMSO, anhydrous, 99.9 %) and N,N-Dimethylformamide (DMF, anhydrous, 99.8 %) were obtained from Sigma-Aldrich. Ethanol (EtOH, 200 proof, anhydrous) was obtained from Decon. All water used was purified to 18 MΩ and passed through a 0.1-mm filter. Metaraminol (free base) and meta-Hydroxyephedrine (*m*-HED) hydrochloride were obtained from ABX. Sodium chloride (NaCl, USP) and ammonium acetate (NH_4OAc, HPLC grade) was purchased from Fisher Chemical. Millex-GV sterile filters with a 0.22-µm pore size were purchased from Millipore Sigma.

Analytical Methods - Radio high-performance liquid chromatography (radio-HPLC) chromatograms for quality control were registered using an 1100 Series HPLC system (Agilent Technologies) equipped with a GabiStar flow-through gamma detector (Raytest) and a 4.6 x 250 mm Aqua 5u C18 125Å column (Phenomenex). Data acquisition and processing was performed using Gina Star 6 software (Raytest). Elution was performed at a constant flow rate of 1 mL/min with 10% EtOH in NH4OAc solution. Detection wavelength was 280 nm.

Semi-preparative HPLC was performed with the TRACERlab FX2 C (GE Healthcare) equipped with a S 1122 HPLC pump (Sykam) and a VP 250/10 NUCLEOSIL 100-5 C18 Nautilus column (Macherey-Nagel). Elution was performed at a constant flow rate of 4 mL/min with 5% EtOH in isotonic 25 mM NH_4OAc (NaCl added for isotonicity). Storage solution for the column was 70% EtOH in water.

Radiosynthesizer Setup - Before each run, the TRACERlab FX2 C synthesizer was checked for leaks in the gas reaction circuit and the reaction vessel. The HPLC purification column and the fraction collection lines were rinsed with 5% EtOH in isotonic 25 mM NH_4OAc. The reaction vessel was charged with metaraminol (0.7 mg, 4.2 µmol) dissolved in DMF:DMSO = 3:1 (320 µL). Vessel 3 was charged with 5% EtOH in isotonic 25 mM NH_4OAc (1.78 mL). Before delivery of the activity, the methane oven was conditioned with a flow of H_2 (50 mL/min) for 20 min at 350 °C.

Synthesis Protocol - $[^{11}C]CO_2$ was produced by the $^{14}N(p,\alpha)^{11}C$ nuclear reaction in an 11 MeV RDS-112 cyclotron (Siemens) at 35 µA using an aluminum target body. The activity was unloaded from the target and pushed through the methane oven at room temperature. Trapped $[^{11}C]CO_2$ was converted into $[^{11}C]CH_4$ with a stream of H_2 (50 mL/min) at 350 °C. $[^{11}C]CH_4$ was trapped in the methane trap under liquid N_2 cooling at -75 °C and purified. Conversion to $[^{11}C]CH_3I$ was achieved by reaction with I_2 at 740 °C in a gas circulating process system. Accumulated $[^{11}C]CH_3I$ was released from the trap upon heating to 190 °C and pushed into the reaction vessel containing the precursor solution at -20 °C. The mixture was reacted for 5 min at 100 °C, cooled to room temperature and diluted with 5% EtOH in isotonic 25 mM NH_4OAc in vessel 3. The mixture was transferred into the HPLC injection loop and purified by HPLC. The product fraction was typically collected from 11 min – 14 min and sterile filtered directly. The final formulation was taken out of the hot cell for quality control and shipment.

Results - $[^{11}C]$*m*-HED was obtained in an activity yield range of 629–1480 MBq (17–40 mCi) within 42 ± 1 min (n = 12). Radiochemical purity was 98.9 ± 1.1 % and molar activity ranged from 7.4–22.2 GBq/µmol (0.2–0.6 Ci/µmol) at the end of synthesis.

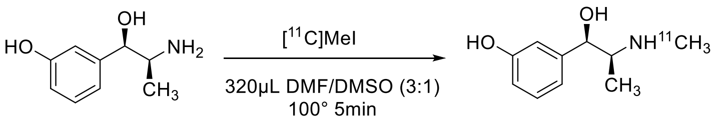

Metaraminol
0.7mg

$[^{11}C]$*m*-HED

Arrhythmogenicity Index

Arrhythmia	Time	Points Awarded
PVC	Baseline	2 (per individual PVC)
PVC (30 or more)	Post NE Injection	4
PVC (10 or more)	Post NE Injection	3
PVC (3 or more)	Post NE Injection	2
PVC (1 or 2)	Post NE Injection	1
Arrhythmia (Bigeminy, Quadrigeminy, Couplets, AIVR, NSVT, etc)	Post NE Injection (10 min)	3 points per occurrence
Arrhythmia (Bigeminy, Quadrigeminy, Couplets, AIVR, NSVT, etc)	Post NE Injection (20 min)	2 points per occurrence
Arrhythmia (Bigeminy, Quadrigeminy, Couplets, AIVR, NSVT, etc)	Post NE Injection (30 min)	1 points per occurrence
Arrhythmia (Bigeminy, Quadrigeminy, Couplets, AIVR, NSVT, etc)	Post NE Injection (30 min +)	0.5 points per occurrence

List of Primers

	Forward	Reverse
GAPDH	CTTTGTCAAGCTCATTTCC	TGCAGCGAACTTTATTGATG
SEMA3A	CGGTGGCTCAATGATCCTAGA	TTTGTCATCTTCAGGGTTGTCACT
NGF	CATAGCGTAATGTCCATGTTGTTCT	CTTCTCATCTGTTGTCAACGC

Immunohistochemistry

Evaluation of noradrenergic nerves in heart sections of mice

Transmural mouse heart sections (100 μm) were stained using primary and secondary fluorescent antibodies. Each section was placed in 400 μl of blocking solution containing 10% Horse Serum (HS), 0.2% Triton X-100, and 0.01 M PBS + 0.02% NaAz solvent for overnight incubation with slight agitation. Next, blocking solution was replaced with a primary antibody solution, containing the same blocking solution from the night before, 1:500 Rabbit PGP 9.5 (Protein Gene Product 9.5), 1:200 Sheep TH (Tyrosine Hydroxylase), and 1:400 Goat NPY (Neuropeptide-Y). After 3 nights of incubation with slight agitation, the sections were washed with 0.01 M PBS twice every hour for a minimum of 5 hours. A secondary antibody solution, containing the same initial blocking solution and 1:200 Anti-Rabbit 488, 1:200 Anti-Sheep Cy3, and 1:200 Anti-Goat 647, was added. Sections were covered to prevent light exposure and incubated for 3 nights with slight agitation. Next, sections were washed with 0.01 M PBS twice every hour for at least 5 hours, all while avoiding light exposure. Under dimly lit conditions soft paint brushes were used to place each section in the middle of a 75 mm long, 25 mm wide, and 1 mm thick glass microscope slide. Tissue mounting media - RIMS (refractive index matching solution) - was added in drops until the sections were covered entirely without excess media and an 18 mm x 18 mm x 0.25 mm coverslip was placed over the tissue mounting media covering the tissue. Slides were labeled, and taken for immediate imaging after a 20-minute drying period (in the dark).

A Zeiss LSM-880 confocal laser microscope (Oberkochen, Germany) was used to image the murine cardiac tissues. The following laser settings were used: 488 - PGP 9.5 (Green), 562 (Cy3) - TH (Red), 647 -NPY (Blue). Whole tissues were imaged at 10x and stitched together using 'tile scan'. A representative area of ~ 20 μm thickness was designated to the Z-stack. All images were exported as .CZI files and converted to Maximum Intensity Projections (MIP) for subsequent processing.

Evaluation of noradrenergic nerves in sections of human left ventricle

Paraffin sections (5 μm) through left ventricular free wall were obtained from archived tissue collected at autopsy from normal hearts and hearts with DCM. Slides were coded to maintain investigator blinding during tissue processing and microscopic evaluation. Sections were deparaffinized, hydrated, treated for antigen retrieval (1 mM EDTA, pH 8, 40 min at 92-94° C), and stained for the noradrenergic marker, tyrosine hydroxylase (TH) at room temperature using the ABC immunohistochemistry method (Rabbit ABC-HRP Kit, PK-4001, Vector Labs). Briefly, slides were rinsed with PBS (pH 7.3), incubated for 10 min in PBS containing 0.4% Triton X-100 and 0.5% bovine serum albumin (BSA), treated for 15 min with 1.0% H_2O_2 in PBS, rinsed an additional time with PBS and incubated 10 min in PBS containing 0.4% Triton X-100 and 0.5% BSA. Slides were then placed in an incubation box and covered with blocking buffer (PBS containing 1% BSA, 0.4% Triton X-100, and normal goat serum). After 2 hours, the blocking buffer was replaced with fresh blocking buffer containing the primary antibody (rabbit anti-TH, Pel-Freez, Cat. No. P40104-150, 1:500 dilution) and incubated overnight. Sections were washed with PBS and PBS containing 0.5% BSA followed by a two-hour incubation in biotinylated secondary antibody (1:200 dilution) from the kit. Slides were washed again, before a 1.5-hour incubation with the ABC reagent from the kit. Slides were next washed for 20 min in 50 mM Tris buffer (pH 7.6) before treatment for 1-10 min with the chromogen (Vector ImmPACT VIP Kit, SK4605) to visualize localization of TH (purple reaction product). Slides were washed, dehydrated and cover glasses attached using Cytoseal XYL (Thermo Scientific Cat. No. 8312-4). Labeled sections were viewed and digital images collected with an Olympus BX41 microscope equipped with an Olympus DP74 digital camera and cellSens software (Olympus America Inc., Center Valley, PA). Two sections from each sample were viewed to determine the presence of noradrenergic nerves in the subepicardium, midwall, and subendocardium, and samples were assigned to the normal or DCM group based on observed innervation pattern. Representative images for each group were collected for figure construction.

List of Antibodies

Primary Antibody	Concentration	Source
Protein Gene Product 9.5 (PGP 9.5)	1:500	Polyclonal Rabbit Antibody. Abcam. San Francisco, CA. ab108986.
Tyrosine hydroxylase (TH)	1:200	Polyclonal Sheep Antibody. EMD Millipore. Darmstadt, Germany. ab1542.
Tyrosine hydroxylase (TH)	1:500	Polyclonal Rabbit Antibody. Pel-Freez. P40104
Neuropeptide- Y (TH)	1:400	Polyclonal Goat Antibody. Novus. Centennial, CO, USA. NBP1-46535.
Vesicular acetylcholine transporter (VAChT)	1:200	Polyclonal Rabbit Antibody. Synaptic Systems Goettingen, Germany. 139-103.

Secondary Antibody	Concentration	Source
Alexa Fluor 488- conjugated AffiniPure Donkey Anti-Rabbit	1:200	Polyclonal Rabbit Antibody. Jackson Immunoresearch Laboratories, Inc. West Grove, PA, USA. 711-545-152
Cy3 conjugated AffiniPure Donkey Anti-Sheep	1:200	Polyclonal Rabbit Antibody. Jackson Immunoresearch Laboratories, Inc. West Grove, PA, USA. 713-165-147.
Cy3 conjugated AffiniPure Donkey Anti-Rabbit	1:200	Polyclonal Rabbit Antibody. Jackson Immunoresearch Laboratories, Inc. West Grove, PA, USA. 711-165-152.
Alexa Flour 647 AffiniPure Donkey Anti-Goat IgG	1:200	Polyclonal Goat Antibody. Jackson Immunoresearch Laboratories, Inc. West Grove, PA, USA. 705-605-147.

Long-Axis Heterogeneity Score

Ratio	Score
Region with ratio of transmural gradients between 1.5-1.99	1 point / region
Region with ratio of transmural gradients between 2.0-2.99	2 points / region
Region with ratio of transmural gradients 3 and above	3 points / region

Myocyte models

The human ventricular action potential model by O'Hara et al was used (1). For normal electrophysiology, control values for the O'Hara model parameters were used with G_{Ks} = 0.01 mS/µF given the small control value. Endocardial and epicardial differences in conductance parameters were taken from the original O'Hara model. For heart failure electrophysiology, changes in ionic currents and SERCA uptake were made according to the experimental ranges detailed in Elshrif et al (2), including specific heart failure remodeling for endocardial versus epicardial cells. (See table below for specific parameters.) A representative action potential for both normal control electrophysiology and heart failure electrophysiology is shown in Figure 7.

Cardiac tissue and anatomical ventricle modeling

The 1D cable was modeled with the following partial differential equation for voltage (V) as

$$\frac{\partial V}{\partial t} = -\frac{I_{ion}}{C_m} + D\frac{\partial^2 V}{\partial x^2}$$

(1)

and the isotropic 2D tissue as

$$\frac{\partial V}{\partial t} = -\frac{I_{ion}}{C_m} + D\left(\frac{\partial^2 V}{\partial x^2} + \frac{\partial^2 V}{\partial y^2}\right)$$

(2)

where C_m is the membrane capacitance and was set to C_m=1 µF/cm^2, and *D* is called the diffusion constant which is proportional to the gap junction conductance. *D*=0.0005 cm^2/ms was used. A time-adaptive Euler method with D*x*=D*y*=0.015 cm and D*t*=0.01-0.10 ms was used for numerical integration of Eqs. 1 and 2.

The human anatomical ventricle model was adapted from our previous study Liu *et al.* (34), originally based on a model developed by Ten Tusscher et al (3,4). In brief, the governing equation for V is

$$\frac{\partial V}{\partial t} = -\frac{I_{ion}}{C_m} + \nabla \cdot \widetilde{D}\, \nabla V$$

(3)

where $\widetilde{D}$ is the diffusion tensor describing the fiber directions in the ventricles (3). Endocardial and epicardial layers were created by defining an equidistant boundary between the epicardial and endocardial surfaces. This boundary was calculated using a fast-marching method as implemented in the scikit-fmm Python module. M-cells were not simulated for simplicity and due to the ongoing debate on their existence. The Purkinje network model was unchanged from our previous study (34) and based on the action potential model by Stewart *et al*. (5). Pacing was initiated from the AV-node through the His-Purkinje system.

Pseudo-ECGs were calculated for 1D and 2D tissue and the human whole-ventricle model using the following formula:

(4)

$$\Phi_e \propto \iiint -\nabla V \cdot \nabla \frac{1}{R}\, dxdydz$$

where $R = \sqrt{(x-x_0)^2 + (y-y_0)^2 + (z-z_0)^2}$ and (x_0, y_0, z_0) is the position of the ECG electrode. Note that for the anatomical ventricle simulations, P-waves are absent since the atria are not modeled.

Beta adrenergic response

The response to beta adrenergic input was primarily simulated by an increase in L-type calcium current $I_{Ca,L}$, similar to our previous study (34). The sympathetic surge was simulated using a ramp protocol for P_{Ca} as shown in Eq. 4 and Figure 7B,

$$P_{Ca} = \begin{cases} P_{Ca,base} + \beta \cdot P_{Ca,low} & t < 5s \\ P_{Ca,base} + \beta \cdot \left(P_{Ca,low} + \frac{t-5s}{25s} \cdot \left(P_{Ca,max} - P_{Ca,low}\right)\right) & 5s \leq t < 25s \\ \beta \cdot P_{Ca,max} & t \geq 25s \end{cases}$$

(5)

where $P_{Ca,base}$ is the intrinsic level of I_{CaL} even without any sympathetic input, $P_{Ca,low}$ is the additional I_{CaL} from a baseline level of resting sympathetic input, and $P_{Ca,max}$ is the maximum level of I_{CaL} conductance during the surge. The parameter β which represents the percentage of sympathetic activation, which has values between 0.0 (fully denervated) and 1.0 (fully innervated), therefore scaling the $I_{Ca,L}$ ramp response. Different areas of denervation in the tissue would have different β values, and therefore different P_{Ca} levels at the end of the ramp. Tissue heterogeneity in β manifests as heterogeneity of I_{CaL} during the sympathetic surge.

Heart failure computer modeling parameters

As stated in the main methods, the control parameters for the O'Hara *et al*. (1) human ventricular action potential model was used for normal physiology, with G_{Ks} adjusted to 0.1 mS/μF given the small control value. The endocardial and epicardial conductances were also taken directly from O'Hara *et al.* Heart failure parameter changes for endocardial and epicardial tissues were taken from Table 2 in Elshrif *et al.* (2). As these parameter changes were given as a range, the mean value was used except as noted below.

These parameters were changed as follows:

Current	Epicardial change	Endocardial change
I_{Na}	↓62.6%*	↓62.6%*
I_{NaL}	↑93.3%	↑93.3%
I_{to}	↓40.4%	↓50.8%
I_{K1}	↓55.3%	↓55.0%
I_{Kr}	↓45.9%*	↓27.3%
I_{Ks}	↓59.4%	↓57.7%
I_{NaCa}	↑131.4%	↑131.4%
I_{NaK}	↓40.2%	↓40.2%
SERCA	↓41.3%	↓41.1%

* For the anatomical ventricle simulations, I_{Na} was reduced by 20% due to conduction issues at the full 62.6%, and I_{Kr} in the epicardium was reduced by 35.0% due to excessive repolarization with 45.9%. These values are still within the ranges of experimental observations as detailed in Elshrif et al.

Supplementary References

1. O'Hara T, et al.. Simulation of the undiseased human cardiac ventricular action potential: model formulation and experimental validation. *PLoS Comput Biol*. May 2011;7(5):e1002061. doi:10.1371/journal.pcbi.1002061
2. Elshrif MM, et al. Representing variability and transmural differences in a model of human heart failure. *IEEE J Biomed Health Inform*. Jul 2015;19(4):1308-20. doi:10.1109/JBHI.2015.2442833
3. Ten Tusscher KH, et al. Organization of ventricular fibrillation in the human heart. *Circ Res*. Jun 22 2007;100(12):e87-101. doi:10.1161/CIRCRESAHA.107.150730
4. Vandersickel N, et al.. Perpetuation of torsade de pointes in heterogeneous hearts: competing foci or re-entry? *J Physiol*. Dec 1 2016;594(23):6865-6878. doi:10.1113/JP271728
5. Stewart P, et al. Mathematical models of the electrical action potential of Purkinje fibre cells. *Philos Trans A Math Phys Eng Sci*. Jun 13 2009;367(1896):2225-55. doi:10.1098/rsta.2008.0283
